# Supplementary material for: Synthesis of Nitro- and Acetyl Derivatives of 3,7,10-Trioxo-2,4,6,8,9,11-hexaaza[3.3.3]propellane
Source: Materials (Basel). 2022 Nov 23;15(23):8320. doi: 10.3390/ma15238320 (PMC9740836; doi:10.3390/ma15238320)
Supplement: Supplementary file 1 [file materials-15-08320-s001.zip › materials-2012423-supplementary.pdf]

energies

MDPI

Review

Major Challenges towards Energy Management and Power Sharing in a Hybrid AC/DC Microgrid: A Review

Sohail Sarwar *, Desen Kirli, Michael M. C. Merlin and Aristides E. Kiprakis

School of Engineering, University of Edinburgh, Faraday Building, King's Buildings, Mayfield Road, Edinburgh EH9 3JL, UK; desen.kirli@ed.ac.uk (D.K.); michael.merlin@ed.ac.uk (M.M.C.M.); aristides.kiprakis@ed.ac.uk (A.E.K.)

* Correspondence: s2124143@ed.ac.uk

Abstract: A fundamental strategy for utilizing green energy from renewable sources to tackle global warming is the microgrid (MG). Due to the predominance of AC microgrids in the existing power system and the substantial increase in DC power generation and DC load demand, the development of AC/DC hybrid microgrids (HMG) is inevitable. Despite increased theoretical efficiency and minimized AC/DC/AC conversion losses, uncertain loading, grid outages, and intermittent complexion of renewables have increased the complexity, which poses a significant threat toward system stability in an HMG. As a result, the amount of research on the stability, management, and control of HMG is growing exponentially, which makes it imperative to recognize existing problems and emerging trends. In this survey, several strategies from the most recent literature developed to address the challenges of HMG are reviewed. Power flow analysis, power sharing (energy management), local and global control of DGs, and a brief examination of the complexity of HMG's protection plans make up the four elements of the review technique in this article. During critical analysis, the test system employed for validation is also taken into consideration. A comprehensive review of the literature demonstrates that MILP is a frequently employed technique for the supervisory control of HMG, whereas tweaking bidirectional converter control is the most common approach in the literature to achieve efficient power sharing. Finally, this review identified the limitations, undiscovered challenges, and major hurdles that need to be addressed in order to develop a sustainable control and management scheme for stable multimode HMG operation.

Keywords: hybrid AC/DC microgrid; power sharing; interlinking converter control; energy management

Citation: Sarwar, S.; Kirli, D.; Merlin, M.M.C.; Kiprakis, A.E. Major Challenges towards Energy Management and Power Sharing in a Hybrid AC/DC Microgrid: A Review. *Energies* **2022**, *15*, x. https://doi.org/10.3390/xxxxx

Academic Editors: Ignacio Hernando-Gil and Ionel Vechiu

Received: 27 September 2022
Accepted: 18 November 2022
Published: 23 November 2022

Publisher's Note: MDPI stays neutral with regard to jurisdictional claims in published maps and institutional affiliations.

Copyright: © 2022 by the authors. Licensee MDPI, Basel, Switzerland. This article is an open access article distributed under the terms and conditions of the Creative Commons Attribution (CC BY) license (https://creativecommons.org/licenses/by/4.0/).

1. Introduction

While the conventional power grid was designed for centralized control and generation from fossil fuels, due to green energy initiatives and net-zero carbon emission goals, the penetration of distributed renewable energy generation (i.e., from wind and solar energy) has drastically increased in the past few decades [1]. According to [2,3], the production and consumption of local energy are cheap, efficient, and reliable, particularly for remote and off-grid applications. The "Three Ds," or "decarbonize, decentralize, and democratize," have emerged as a key focus for the entire electrical system in order to lower carbon emissions, end the utility grid's monopoly, provide power to even remote communities by enhancing infrastructure, and put more of an emphasis on the grid's resilience and dependability [4,5]. Hence, research in the field of "microgrids" explored the benefits and drawbacks of operating these smart and autonomous networks with high distributed energy resources (e.g., solar), which induce bidirectional power flow on a grid that was designed as a one-way connector between centralized generation and distributed consumers [6,7].

Several microgrid configurations, such as AC, DC, and hybrid AC/DC based on power type [8,9] as well as grid supportive, grid-feeding, and grid-forming microgrids [10,11] based on power converter type, have been investigated. The advantages of AC microgrids include minimal power electronic interface and reasonably straightforward integration with the current power network. However, the need for DC microgrid development is pushed by the significant rise in DC load demand and the DC network's self-fault-ride through capability [12]. Consequently, a hybrid AC/DC microgrid can achieve the benefits of both. Additionally, HMG will reduce power conversion losses, reduce the number of power converters needed, and eliminate the requirement for a separate DC line. Despite increased theoretical efficiency and minimized AC/DC/AC conversion losses, uncertain loading, grid outages, and intermittent complexion of renewables have increased the complexity, which poses a significant threat toward system stability in an HMG. An exponential rise in research in this field has shown how important it is to recognize current issues and future trends. Therefore, in order to realize the HMG concept, considerable attempts have been made to investigate current methodologies.

The development and integration of microgrids enhance the flexibility and performance of the grid using a variety of control algorithms. A critical investigation of control techniques for converters associated with RESs was provided in [13–16], whereas [17,18] discussed multiple solutions proposed in the literature for adaptive protection settings in grid-connected and islanded HMG. The uncertainty of renewables significantly affects the power delivery and the transients caused by them and their remedies are studied in [19]. The transients due to renewable uncertainty and power converters disturb the power quality and voltage and frequency stability, which has been investigated in [20,21]. All of the discussed issues are vital and given due importance in the literature. Power/energy management and load management of any power network and especially for HMG are critical aspects due to the complex control of power converters. It is evident from this analysis that the management of HMG has not been reviewed. Hence, this article focuses on load and energy management along with power sharing in an HMG. The existing literature is critically analyzed and summarized to provide the recent methods implemented and future trends in this important field.

Review Methodology

Various attempts have been made to examine the current literature because it is an emerging subject in the power system as a result of the exponential rise in the generation of DC power from solar energy. In order to distinguish between our approach and their strategy of a primary topic (hybrid microgrid), but different sub-problem (managing the power and energy), these review strategies are discussed in Section 1. More than half of the analysis in all of the review publications discussed above has been conducted for methodologies developed for a specific AC or DC microgrid. As a result, this study uses Research Rabbit to filter and analyze approaches developed specifically for hybrid microgrids. Figure 1 illustrates the review process and data collection.

An organized search and review process was used to assess how power and energy are managed in hybrid microgrids for our study. In order to find relevant material, the terms "hybrid microgrid" and "energy management" or "power sharing" were entered into search engines (Google Scholar and Web of Science). The literature containing the aforementioned keywords in its title, abstract, and keywords was filtered and examined. This produced 36 peer-reviewed publications that were the most appropriate after being filtered for relevance. These papers served as the tool's input. Based on the citation information, Research Rabbit correlates the input with various related work. Based on the title and abstract, irrelevant papers were filtered out of the dataset, which produced a handful of articles. The critical analysis of these articles is discussed in the upcoming sections.

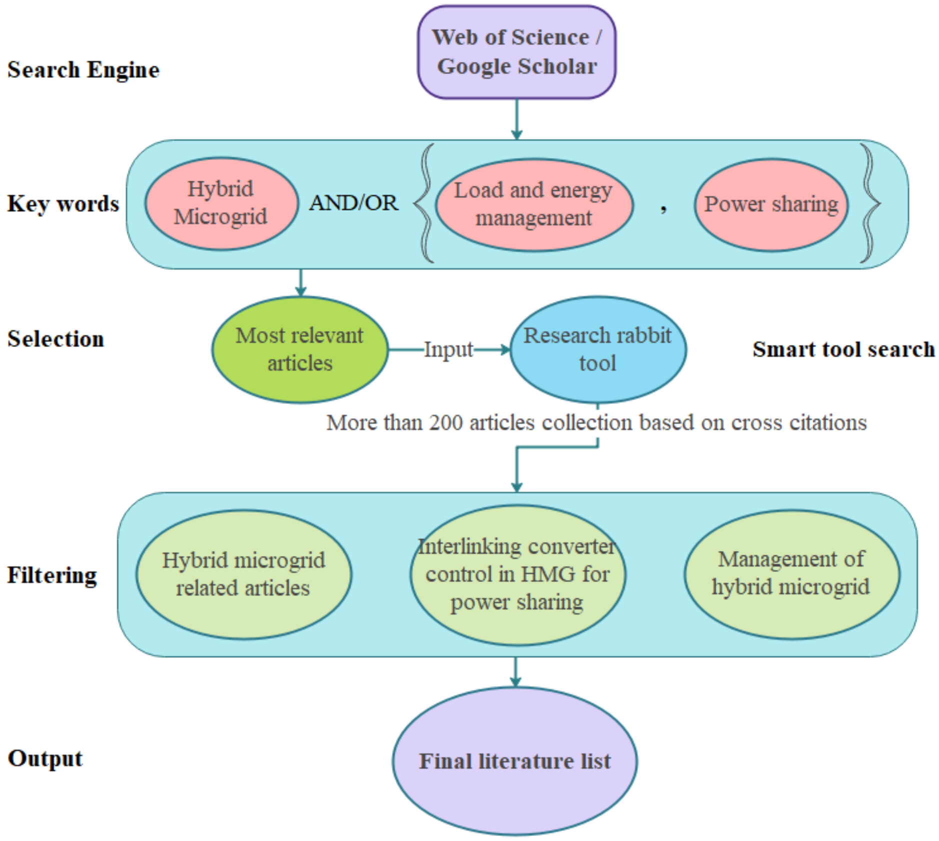

Figure 1. Methodology of data collection for this review.

The primary objective of a stable hybrid microgrid can be categorized into multiple sub-objectives. A brief review of the shortlisted literature resulted in the distribution of various objectives, which are displayed in Figure 2. It is evident that the most extensively studied objective is bidirectional power sharing to support adjacent subgrids in case of contingency, as expected (one-fourth of the literature reviewed). Therefore, more detailed analysis and discussion of it, as well as other objectives, will be covered in the following sections. The four components of the review methodology in this article are power flow analysis, power sharing (energy management), local and global control of DGs, and a brief inspection of the complexity of HMG's protection strategies. The applicability of the techniques developed to achieve these objectives along with their limitations is detailed in the next sections.

This paper is structured as follows. Section 2 highlights the operational challenges of hybrid microgrids. Section 3 discusses power flow analysis methodologies and issues. Section 4 provides a deep discussion of load and energy management. Section 5 reviews and summarizes the adaptive protection and local management tactics of RESs. Section 6 presents the findings and contribution of this work with recommendations for future work. Section 7 concludes the paper by summarizing the findings from this extensive review.

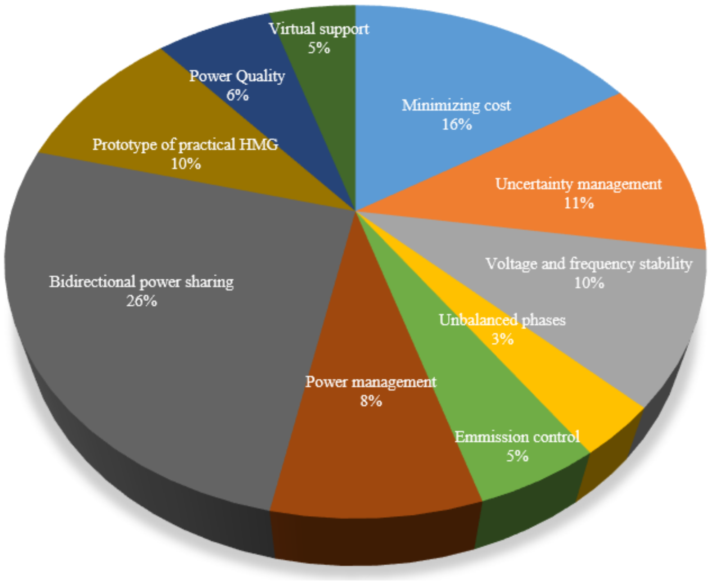

Figure 2. Distribution of multiple objectives in literature.

2. Hybrid Microgrids

Combining AC and DC microgrids to avoid frequent AC/DC/AC conversion is an alternative option. A significant set of AC/DC powered devices connected to their respective common busses and interfaced through power converters can be deployed as a hybrid microgrid. Multiple topologies/configurations for hybrid microgrids such as AC-coupled, DC-coupled, and AC decoupled have been studied and detailed in [22,23]. The significance of a hybrid microgrid is that it can serve local energy demands via DGs while also connecting AC and DC loads to their respective supplies, reducing conversion losses [24]. A hybrid AC/DC microgrid shown in Figure 3, from an operational standpoint, is a gateway for future power distribution systems. Furthermore, by combining AC and DC microgrids to build a hybrid microgrid, greater energy consumption standards, higher dependability, enhanced power quality, and system stability can be accomplished [25]. The major benefits of an HMG can be listed as:

- Integration of new generating or consumer points.
- No requirement of synchronizing for additional ESS and PVs by direct connection to the DC bus.
- Voltage transformation is significantly reduced [26].
- A reduced number of converters and losses ensure economic feasibility [27].

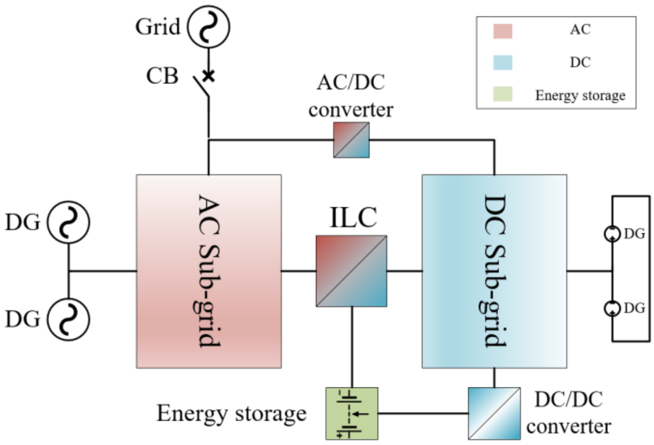

Figure 3. Layout of a hybrid microgrid.

The dynamics of power system operation for hybrid microgrids have entirely changed, increasing the complexity. Particularly in an isolated/standalone mode of operation, a hybrid microgrid is more vulnerable to instability. The main disadvantages of HMG are the compromised protection settings, fragile stability, and control complexity when compared to AC microgrids. Despite the fact that the HMG has various advantages on paper, further research is needed to address the disadvantages and take over as a substitute for conventional power systems in the future.

Operational Challenges of Hybrid Microgrids

In HMG, power conversion losses and the number of power electronic interfaces are minimized. However, operational stability is at stake due to increased complexity. The shift in operational dynamics due to RESs creates multiple challenges related to design, stability, protection, and control, which are graphically presented in Figure 4. Therefore, utilities and researchers have invested in hybrid microgrid R&D in order to optimize its operation. Although distributed generation has enhanced the reliability of the power network, the system's stability with multiple microgrids is in jeopardy. A hybrid microgrid in islanding mode is more vulnerable to unbalanced generation and load demand. As a result, energy and load management have become critical components of hybrid system stability in order to avoid system blackouts [28,29].

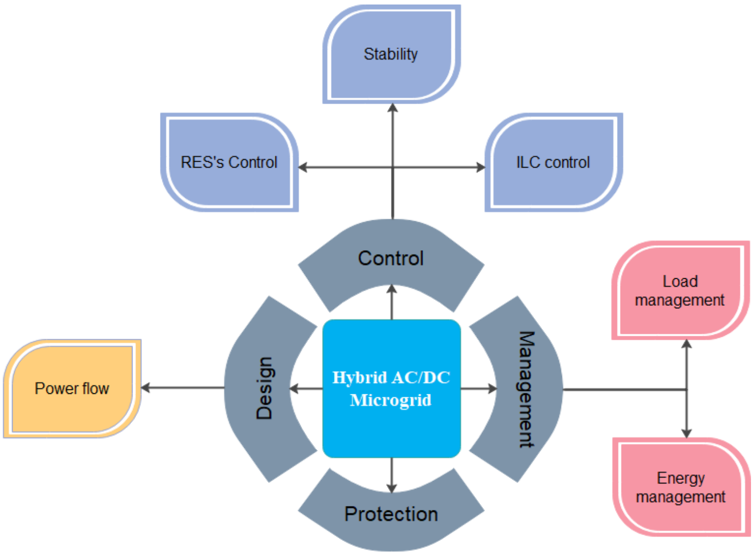

Figure 4. Issues of an operational hybrid microgrid.

In a hybrid microgrid, the bidirectional power flow between AC and DC subgrids adds to the complexity. When planning a hybrid microgrid, the power flow analysis is not as straightforward as it is with conventional power systems [30]. The most challenging and innovative part is the control of the bidirectional converter [14]. The local control of RESs also needs to be improvised to stabilize the terminal parameters [14]. Similarly, the system's protective coordinates may fail due to a variation of fault current levels, despite the fact that the system's protection settings are optimal [17]. As a result, the practical deployment of a hybrid AC/DC microgrid remains debatable. A significant amount of research has been conducted to resolve these challenges, which is critically examined in the following sections.

3. Power Flow Analysis

In the design stage of HMGs, power flow simulations are significant for analyzing a variety of issues such as voltage stability, DGs and interlinking converter placement and sizing, and contingency/fault analysis. It also aids in the monitoring of variables, i.e.,

voltage, frequency, and line thermal limitations. Furthermore, the power flow can be an important instrument in real-time HMG operation for observing power loss, cost optimization, and energy management, among other things [30,31]. Table 1 summarizes all of the solutions for the HMG's power flow, which will be addressed in detail later. The interconnection of AC and DC subgrids adds to the complexity of solving power flow for a hybrid microgrid. A second-order cone-programming-based power flow solution is presented in [32] for hybrid microgrids. However, only the grid-connected mode is investigated in this study. Because only voltage source converter-based DC loads/busses are considered, multiple types of DGs may render this solution invalid. Another viable solution for the power flow of a hybrid microgrid is to equalize the normalized values of AC frequency and DC voltage. To overcome the previously mentioned difficulty of microgrid multiple mode operation, the Newton–Raphson (NR) approach is used sequentially to design a new algorithm [33]. In a hybrid microgrid, higher R/X values have a significant impact on power flow calculations. To minimize this effect of highly resistive networks, the Levenberg–Marquardt algorithm is used in [34] where the power flow is visualized for a basic six-bus AC/DC network with a fifteen-fold increase in R/X value. The calculation of the Jacobian matrix at each iteration for the above-discussed techniques increases the computational time. A forward–return forward–backward sweep strategy for branch-based sequential power flow is presented in [35]. A new model is developed in this study to reflect the DC subgrid and its effect on the AC subgrid and vice versa to tackle the convergence of the algorithm and uncertainty of renewables. Due to repeated loops that make the algorithm complicated and time consuming, the sequential solution of the AC/DC subgrid power flow may raise questions about its convergence.

Table 1. Review table of power flow analysis for a hybrid microgrid.

Reference	Mode of Operation GC/ISO	Absence of Slack Bus	Unbalanced Operation of Subgrids	Parallel Operation of ILCs	Load Variation and Types	Uncertainty of Renewables	Convergence of Algorithm	Stability
[30]	Both	✓	✗	✗	✗	✗	✓	✗
[32]	GC only	✗	✗	✗	✗	✗	✗	✗
[33]	Both	✓	✗	✗	✗	✗	✗	✗
[34]	Both	✓	✗	✗	✗	✗	✗	✗
[35]	ISO only	✓	✗	✗	✓	✓	✓	✗
[36]	GC only	✗	✗	✗	✗	✗	✓	✗
[37]	GC only	✗	✗	✗	✗	✗	✓	✗
[38]	Both	✓	✓	✗	✗	✗	✓	✗
[39]	Both	✓	✗	✓	✓	✗	✓	✓
[40]	ISO	✓	✗	✓	✗	✗	✓	✓
**[41]**	**ISO**	✓	✓	✓	✗	✗	✓	✗

A unified method for HMG's power flow may be adopted as an alternative approach to the sequential solution [36]. In this research, a unified power flow method for a hybrid microgrid with multiple AC subgrids and a tiny DC subgrid is developed. However, modeling power converter equations in a saturated AC grid was merely a basic idea to unify the power flow analysis. Another unified strategy is offered in [37], this time based on the Newton trust-region (NTR) concept. Multiple load types and DG types are studied for small and large systems in a unified approach for load flow analysis specially designed to analyze the absence of a slack bus [38]. Voltage and frequency variation was not given due consideration for an islanded microgrid. Another generic power flow approach based on the NTR method, focusing on voltage and frequency stability and the parallel operation of ICs for power sharing, is proposed in [39]. A modified Jacobian matrix-based NR method is employed in a novel unified power flow methodology (centered on multiple AC and DC subgrids) [40]. AC frequency and DC voltage are coupled incorporating ILC

droop management to improve power sharing amongst the subgrids. Comprehensive research on hybrid microgrid power flow analysis was presented in [41]. The implicit Z_{BUS} method is employed to tackle this power flow problem in a multi-grounded imbalanced hybrid microgrid. This generic method addresses a variety of challenges in hybrid microgrid power flow analysis, including unbalanced operation, bipolar DC, and multi-grounded systems. However, a critical examination of this study and all of the techniques summarized in Table 1 reveals that in an unbalanced multi-grounded hybrid microgrid with bipolar DC, this problem still necessitates a method to simultaneously investigate load models, voltage, and frequency stability due to load variation and renewables uncertainties.

4. Load and Energy Management (Power Sharing)

One of the primary challenges for the stable operation of a hybrid microgrid, among many others, is load and energy management for power stability and reliability. Although conventional droop control of DGs in AC and DC microgrids can lead to a stable operation under normal circumstances, uncertainties and unplanned events of generation loss and load variation lead to system failure (blackout) [42]. In such extreme circumstances, a well-designed power management strategy is essential for the steady operation of microgrids (power systems). Hence, it can be extrapolated that power sharing in a hybrid microgrid is more difficult and complex than droop control in a conventional power system. Even having an intelligent communication link between DGs (spread on AC and DC subgrids) to follow power sharing is ineffectual under extreme conditions [43]. Modifying the ILC control to follow the intelligent power flow is a basic solution for this problem [44]. Forecasting generation and load levels to schedule power sharing for the next day is another way to deal with these uncertainties and load swings [45]. Additional energy storage systems in HMG aid the complexity levels and can be utilized to stabilize the system with a grid-supporting DC–DC converter [46]. Multiple power sharing and energy management schemes have been developed in the literature, which will be discussed in detail in subsequent sections. Figure 5 shows an overview of multiple energy management strategies to achieve optimal power sharing in multimode HMG.

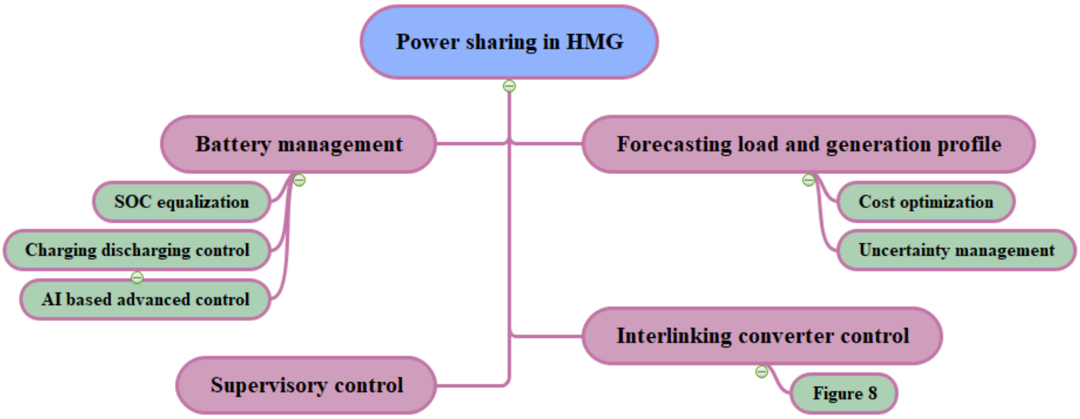

Figure 5. Load and energy management in hybrid microgrid.

4.1. Forecasting the Load and Generation Profile

Smart pricing and maximizing renewable energy to decarbonize the planet are the trending fields in the developed world. Distributed generation has modernized the power system with an option to prioritize renewables for lowering the overall cost. Hence, forecasting the next day's generation and with adequate knowledge of demand response is utilized in the power-sharing scheme for the next day [45]. In [45], the MILP model is used

to optimize the power flow and operational costs based on the predicted generation and load levels for the next 24 h, with batteries and electric vehicles serving as backup during unbalance. According to the authors of [47], the Firefly algorithm, when combined with the alternate direction method of multipliers (ADMM), can produce a superior cost optimization for the next day's anticipated power sharing. The validation of this approach, on the other hand, reveals that it is a specified solution for introducing DC-based RES into the IEEE 33-bus AC distribution system. In [48], it is claimed that the ADMM-based strategy can accomplish effective energy management where the HMG energy management problem is modeled using mixed-integer quadratic programming (MIQP). However, one significant drawback of this method is that renewable uncertainty is not taken into account. Another forecasting-based power balancing scheme in [49] maximizes ESS usage. The authors in [49] claim that this particle swarm optimization (PSO)-based fuzzy logic controller uses ESS to respond to fluctuations and prediction errors. The application of double uncertainty optimization theory may be used to minimize the operational cost by managing the multiple parallel converters for day-ahead electricity scheduling. On the other side, a cost-minimization strategy based on non-cooperative gaming theory is developed and evaluated for a real network in Singapore [50]. This gaming-based technique also introduced a new conversion loss cost for minimizing the conversion losses. Although these AI-based advanced schemes provide an improved cost-effective solution, power sharing management is still questionable. A modified forecasting-based power-sharing approach to overcome the uncertainties is proposed in [29] to realize the power-sharing with multiple predicted errors in forecasting generation profiles. Another risk-based uncertainty optimization is proposed in [51], to optimize the cost and mitigate forecasting errors. This technique converts multiple non-linear uncertainties of interlinking converter and renewables to a MILP-based linear problem. Although this non-cooperative game theory-based scheme [29] and piecewise linearization-based approach [51] model the uncertainties to a certain accuracy, this complicated control in the former does not evaluate its impact on local DG and ILC control, which is a low-level control. The latter one focuses mainly on minimizing the cost instead of diminishing the real-time power surges. However, unforeseen outages and real-time load fluctuations are not addressed.

A dynamic decision maker approach to update the day-ahead control signals for the microgrid entities can minimize the cost considering renewable uncertainties [52]. This MILP-based scheme is problem-specified solution for a remote HMG with the main objective to ensure water supply to residents of the region. Another objective for HMG in addition to cost minimization is an eco-friendly operation by reducing emissions. Such a fuzzy-based multi-objective cost and emission minimization scheme is developed in [53] for a projected load profile of the next day. Another innovative scheme for predicted power profiles is proposed in [54] based on a modified crow search algorithm. It is claimed that, although the designed technique has a somewhat higher operational cost than other deterministic schemes such as GA and PSO, this is realistic and practical when considering the uncertainties of renewables. Supervisory control for HMG in addition to ILC control using MILP is claimed to better manage unintentional solar and wind outages effectively [55]. Additional fault outages are also claimed to be managed by the proposed scheme. Another hierarchal multi-level MILP-based optimization is proposed in [56] for optimal HMG operation. This consensus algorithm, in addition to MG optimization and coordinated optimization among DGs is reported to be capable of managing the power between multiple AC and DC microgrids interconnected through a single ILC. Increased renewable penetration disrupts predicted demand and generation profiles due to a higher voltage and frequency instability. A new supervisory control is proposed in [57] to handle such scenarios by updating the predictive uncertainty, with the additional benefit of lowering the cost of generation for droop-based HMG. However, the realistic issue of a negative power imbalance between generation and load is not addressed.

Forecasting the day-ahead profile with multiple techniques discussed above is a better electricity pricing approach. However, the real-world contingencies related to

unforeseen circumstances in generation and load may cause voltage and frequency instability. Therefore, forecasting is not the best option, and a real-time strategy is required to address this problem. The cause of such a problem is mainly real-time minimal voltage and frequency fluctuations in a synchronized grid, especially in the isolated state [58].

Table 2. Review table for power management of HMG based on forecasting next-day demand.

Ref	Contribution	Technique Used	Limitation	System for Validation
[27]	Cost optimization with minimized mode switching	MILP and mathematical model	Assumed contingency scenarios only	Multiple subgrids with intermittent and dispatchable DGs
[29]	Uncertainty handling by two-stage optimization to realize power management from forecasted curves	Non-Cooperative Game Theory<br>Mathematical programming with complementarity constraints (MPCC)	Complex control that ignores the impact on local control of DGs and ILCs.<br>No solution to the generation and load imbalance	Modified IEEE 33-bus system
[45]	Minimizing cost by forecasting day-ahead hourly demand	MILP optimization to minimize the cost	No real-time validation	Hybrid framework for validation is just like a generation plant with AC and DC units
[47]	Day-ahead forecasting based power sharing to optimize the cost	Firefly algorithm (FA)	Focused only to interface multiple DC RES' with IEEE 33-bus system	IEEE 33-bus system with additional DC generation units as DC microgrid
[49]	Optimizing the net power in microgrid using ESS to overcome the prediction error in load and generation profile	Fuzzy controller based on results of particle swarm optimization results	Quantitative net power balancing without considering real-time operation constraints	Modified DC subgrid with PV and wind source
[50]	Cost optimization	Non-Cooperative Game Theory	No solution to the generation and load imbalance	Cluster of 3 MGS, part of Singapore power network
[51]	Uncertainty modeling for day-ahead power management involving EVs	Piecewise linearization combined with quadratic Newton–Gregory interpolating polynomial technique<br>MILP	Forecasting-based cost optimization only	Microgrid composed of diesel generator, wind, solar, and EVs
[52]	Minimizing cost	MILP optimization	Problem-specified solution	38-bus remote system
[53]	Cost and emission minimizing	Fuzzy logic for two different objectives	Focused only on lowering emissions from a generation unit	6-bus generation dominant HMG
[54]	Optimized realistic cost and energy management	Modified Crow Search Algorithm	Solution for grid-connected system only	Modified IEEE 33-bus system with addition DC busses as DC subgrid
[55]	Power management in PV and wind outages scenario	MILP as supervisory control	Realistic issue of a negative power imbalance is not addressed	PV and wind-based DGS on AC and DC sides, respectively, with local loads
[56]	Optimal power management for grid-connected multiple microgrids (HMG)	MILP optimization	Only grid-connected mode of operation discussed	Multiple AC and DC MGs connected through a single ILC

[57]	Increased stability of voltage for increased penetration of renewable generation	Modified supervisory control based on predicted uncertainties	Prediction-based uncertainties model only	Modified IEEE 33-bus system with additional DC busses and generation
[59]	Minimized operational cost	Double uncertainty optimization theory, fuzzy stochastic optimization	Focused on cost only, stiff grid is assumed to be available always	HMG with PV, wind, diesel generator, and ESS

4.2. Bidirectional Power Converter Control

Forecasting the load and generation profile of a hybrid microgrid made up of multiple AC and DC subgrids for power sharing may fail due to the uncertainty of load and renewables. Hence, power sharing amongst the subgrids using ILC droop control gives a better stability model for HMG [60,61]. Limiting the power exchange across subgrids improves power quality; yet, maximizing DG power at the expense of power quality prevents blackouts due to overloading [62]. However, the system's equilibrium is still disrupted by fluctuations in RES generation and fluctuating loads. For a simple HMG with a DG on each subgrid, a modified DC/AC inverter is developed based on PR and PI controllers as an alternative to traditional d-q control [63]. A real-time lab-based prototype of an HMG is designed in [60] based on frequency droop control to actively share the power in grid-connected mode. This autonomous control scheme suggested that an alternative real-time approach for power sharing is to tweak the ILC control to overcome these minimal fluctuations instead of forecasting profiles. A basic time-based decentralized droop control for HMG control is presented in [64]. Another modified decentralized control is proposed in [58] by modifying droop control with a load damping constant. Contrary to decentralized control, [65] suggested a centralized control scheme to operate multiple converters in parallel for improving the efficiency of converters. Conventional decentralized [58,64], centralized [65], and autonomous droop control-based [60] power-sharing approaches were the foundation for initiating a strong research program in the future to realize the HMG concept. As a result, a practical, multi-level control design for ILC parallel operation is proposed in [66]. Additional secondary and tertiary controls are designed to minimize voltage fluctuations in primary control and enable DC grid connection, respectively. Contrary to the schemes outlined above, a 3D droop control (shown in Figure 6) of ILC has been presented and validated in a lab-based HMG [67]. In addition to voltage and frequency controls, this 3D control of active power droop can meet the active power-sharing demand while incorporating maximum ILC loading, whereas [68] suggests that the droop coefficients of AC and DC DGs can be translated to active powers of respective subgrids. This power calculation, combined with local subgrid load calculations, can be used to build a new local droop coefficient for global power management, including ILC parallel operation. However, all of the techniques discussed did not consider the reactive power management, which is a critical part of HMG.

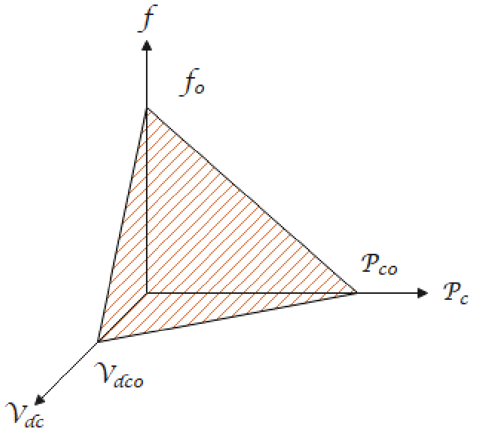

Figure 6. Three-dimensional droop control of ILC (adapted from [67]).

Modifying the converter control by Clarke and Park's transformation can realize the reactive power management through an inverter between AC and DC subgrids [69]. Reactive power compensation based on the d-q control of ILC in a prototype of HMG is proposed in [70]. The results of this reactive power compensation-based control scheme indicate that more complicated and advanced ILC control can improve HMG voltage stability. Another mathematically modified droop control for efficient active and reactive power sharing is proposed in [71]. Power-factor-based calculations of the droop coefficient for active and reactive power control led to an overall improved power sharing under multiple stable scenarios.

In addition to reactive power sharing control from ILC, the droop control mechanism for HMG is not that straightforward, especially for ILC control. Therefore, a new d-q-0 axis control technique is developed in [72], which regulates the V_{dc}^2 rather than V_{dc} to linearize the voltage fluctuations. This scheme claims to have shifted an HMG from grid connected to islanded mode without requiring changes to the control algorithm. However, the power-sharing from the ESS is not optimized, and ineffective discharge causes voltage overshoots in underload conditions. Another d-q-0 axis control-based technique is designed to suppress the circulating currents (missing in [72]) in the parallel operation of power converters [73], which requires a separate control algorithm for islanding and grid-connected mode. The frequency droop control equivalent to Vdc is an alternative for suppressing the circulating currents [74]. The modified droop control for power sharing is based on Equation (1).

$$P_{ILC} = K\left(V_{pu} - f_{ac_{pu}}\right) \quad (1)$$

where V_{pu} is normalized DC voltage compared with PU frequency of AC subgrid for power sharing P_{ILC} between the grids. A control strategy based on an accurate line impedance calculation is proposed in [75]. A small signal is used to estimate the impedance in this scheme, which reduces the dependence on a communication link. This approach may provide better power sharing than conventional control, but voltage regulation and hence reactive power are vulnerable. A modified current control mechanism for ILC (active power sharing) based on the common bus voltage instead of terminal voltages of DGs is presented [76]. However, this technique is only tested on a stable, modest, and balanced microgrid with only six busses, limiting its applicability. In grid-linked mode, ILC control can also be tweaked to improve power quality. The author in [77] proposed a modified ILC version that includes an additional parallel converter with ILC, which aids in the removal of harmonics and is depicted in Figure 7, but may not apply to islanded systems. On the other hand, the authors in [78] recommended using an ESS converter control to operate as a virtual synchronous generator (VSG) to help limit frequency and voltage

fluctuations. However, because VSG is connected to ESS on the AC subgrid and the DC microgrid only has a PV source, the system's applicability is in question.

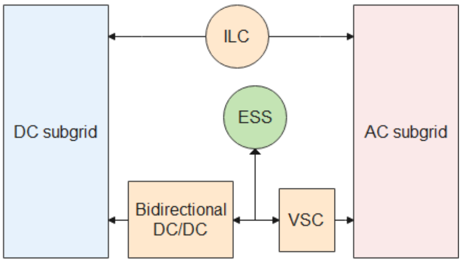

Figure 7. Modified ILC version to increase power quality [77].

The traditional HMG control is straightforward for implementation and adaptation. However, in reality, the slow dynamics [79] and high-frequency fluctuations [80] limit the method's performance. Li et al. in [81] present a modified version of the classic droop control method with a feedforward current control loop for ILC to improve the dynamics of the HMG. The ILC is taught to behave as a converter-based transformer (transfverter) in [82] that manages power flow across subgrids during multiple operational modes of HMG. A redesigned current control method for the ILC is proposed in [83] to improve the dynamic response and robustness to uncertainties and fluctuations in the system. Because the approaches of [82–84] run the ILC in current control mode, the AC subgrid receives only limited frequency and voltage support during transients. Furthermore, the use of a phase-locked loop (PLL) for frequency sensing introduces an unnecessary delay into these systems' ILC control loops. Therefore, a modified voltage-based decentralized control strategy is designed in [85]. The local control of RESs is updated based on the droop coefficient of ILC control in this approach to minimize transients. Although this approach provides smooth power sharing for load disturbance, it has not been validated for an unbalanced generation.

In the literature, there have been various novel control designs for ILC control and power management in an HMG. Such an ANN converter control, combined with fuzzy-based power management for grid-connected mode operation of HMG, is proposed in [86]. The maximum power point (MPPT) of wind and PV-based DC-producing units is tracked by the ANN controller, which supplies local AC and DC loads. Fuzzy-based controllers, on the other hand, manage the power purchased from the grid. A cost and emission minimization approach by modifying the droop coefficient of the power share is proposed in [87]. The active power droop constant m_{pi} is optimized using a hybrid fuzzy-PSO technique considering the uncertainty of renewable generation for a small six-bus HMG. Another scheme in [88] suggests a modified control strategy for effective power-sharing based on input–output feedback linearization (IOFL). According to the authors, this DC bus voltage-based control, with an additional sliding mode controller for current error, achieves better power sharing than traditional droop control. In [89], a model predictive control-based approach is introduced that regulates the most deviated parameter (AC/DC current or voltage) using multivariable optimization. Another study in [90] suggests multiple control strategies to be adopted as a master–slave system for the parallel operation of ILC. A master converter adopts Q-V_{dc} control, while the slaves follow P-Q control for power sharing. However, the validation of this scheme is carried out with a single DC-generating unit connected with multiple AC subgrids, questioning the applicability. An alternative approach to innovative ILC control is designed based on a flying capacitor multi-level converter to improve the AC power quality, while also reducing the filter size [91]. However, this technique is solely intended for grid-connected systems and has been tested on multiple DC-generating units connected to the grid through ILC.

In islanded HMG, hierarchical control of the ILC, comprising primary and secondary control layers based on distributed consensus algorithm [92], can adapt to better power

sharing, especially accurate reactive power sharing. As the algorithm in [92] is specified for islanding operation, a new seamless mode transition method with two independent controls for grid-connected and islanding scenarios is devised in [93]. However, islanding detection is not discussed in detail, which questions the shift of controls from on-grid to off-grid mode. On the other hand, [94] suggested a distributed co-operative secondary control technique for power sharing based on common bus voltage droop management and communication graph of power sharing among DGs. Another error minimization approach based on passivity theory along with a sliding mode controller is proposed to achieve robust power sharing [95]. This scheme compensates for the variable converter impedances through the slide mode controller, which affects the power sharing significantly. In the literature, a new set point weighting iterative learning method-based controller for the ILC's outer voltage control loop is also proposed [96]. DC bus voltage regulation in this scheme dictates that efficient power sharing is achieved. Although these techniques achieve trustworthy power sharing, their effectiveness is tested in a stable HMG (DC generating units synchronized with grid) environment, raising concerns about their adaptation to contingencies and uncertainties.

The presence of an unbalanced load in a real power system disturbs the overall power management and voltage stability. To compensate for this imbalance, a hierarchical distributed control is proposed in [97] in which the interior point method is used to solve this analytical power flow problem for balancing the unbalance load between the phases and increase the load-ability of the system. A very similar approach is also proposed in [98]. The upper layer is intended for simple droop control power management, whereas an event-triggered bottom layer looks after imbalance generation and loads to minimize error and manage a better power flow across the phases for an AC subgrid with single- and three-phase generators. Additionally, the voltage quality of the local DGs and PCC is improved, utilizing a hybrid current and voltage control method based on the proportional-resonant first proposed in [99]. Despite the fact that this system compensates for unbalanced load and generation in grid-connected mode, the upper layer event control is basic droop control, which can be improved to produce better power sharing in islanding and unstable circumstances. A hierarchical method can also aid to stabilize the microgrid's voltage and frequency with an additional secondary control loop [100], along with inner and outer loop controls for moving electricity between the grids. Equations (2) and (3) explain the secondary control of the scheme. However, the control system lacks the solution for unbalanced generation and load in case of contingency and severe fluctuations in voltage stability.

$$\delta f = k_{PF}(f_{MG}^{*} - f_{MG}) + k_{IF}\int(f_{MG}^{*} - f_{MG}) \tag{2}$$

$$\delta V = k_{PV}(V_{MG}^{*} - V_{MG}) + k_{IV}\int(V_{MG}^{*} - V_{MG}) \tag{3}$$

where f and V represent the frequency and voltage of the AC microgrid, respectively.

Voltage and frequency stability is a critical concern for HMG as the power flow between the subgrids increases the transients on the DGs. Hence, an improved scheme to normalize AC frequency and DC voltage is designed to support these parameters [101]. A modified power reference generator aids in enhancing the upper limit of maximum power transfer through the ILC. However, this scheme is validated with ESS as DGs in both subgrids with a lumped load on each side. The presence of actual DGs has a significantly different transient response to power transfer. Another approach in [102] designs a modified droop control that achieves efficient power sharing by utilizing the frequency of ripples (harmonics) in DC at the common DC bus. However, the validation system only has DC DGs. The presence of actual DGs has a significantly different transient response to power transfer.

HMG has the major benefit of reduced AC–DC conversions; however, this infrastructure still requires a considerable number of power converters. A modified UIPC is

modeled for HMG in [103] to reduce the number of power converters. The unified interphase power controller (UIPC) is an alternative to ILC, which connects the AC and DC subgrids directly to the main grid. However, the scheme's efficiency is confined to balanced sub-grids in grid-connected mode only, raising concerns about the technique's applicability. A multiple-surface sliding control approach based on a nonlinear disturbance observer is also provided for stabilizing DC fluctuation owing to photovoltaics. A bidirectional ILC can be coupled in parallel with a unidirectional full bridge (UDFB) rectifier in another novel multiport ILC design to reduce the number of converter switches [104]. A UDFB rectifier is modified to supply variable voltage to various DC distributed loads at the far end. However, it can only be regarded as a problem-specific solution that would require significant modification and validation before being adopted as a general ILC design. A comprehensive review of all the techniques discussed in this section is summarized in Table 3, while Figure 8 provides a pictorial display of some of the important power-sharing strategies in an HMG.

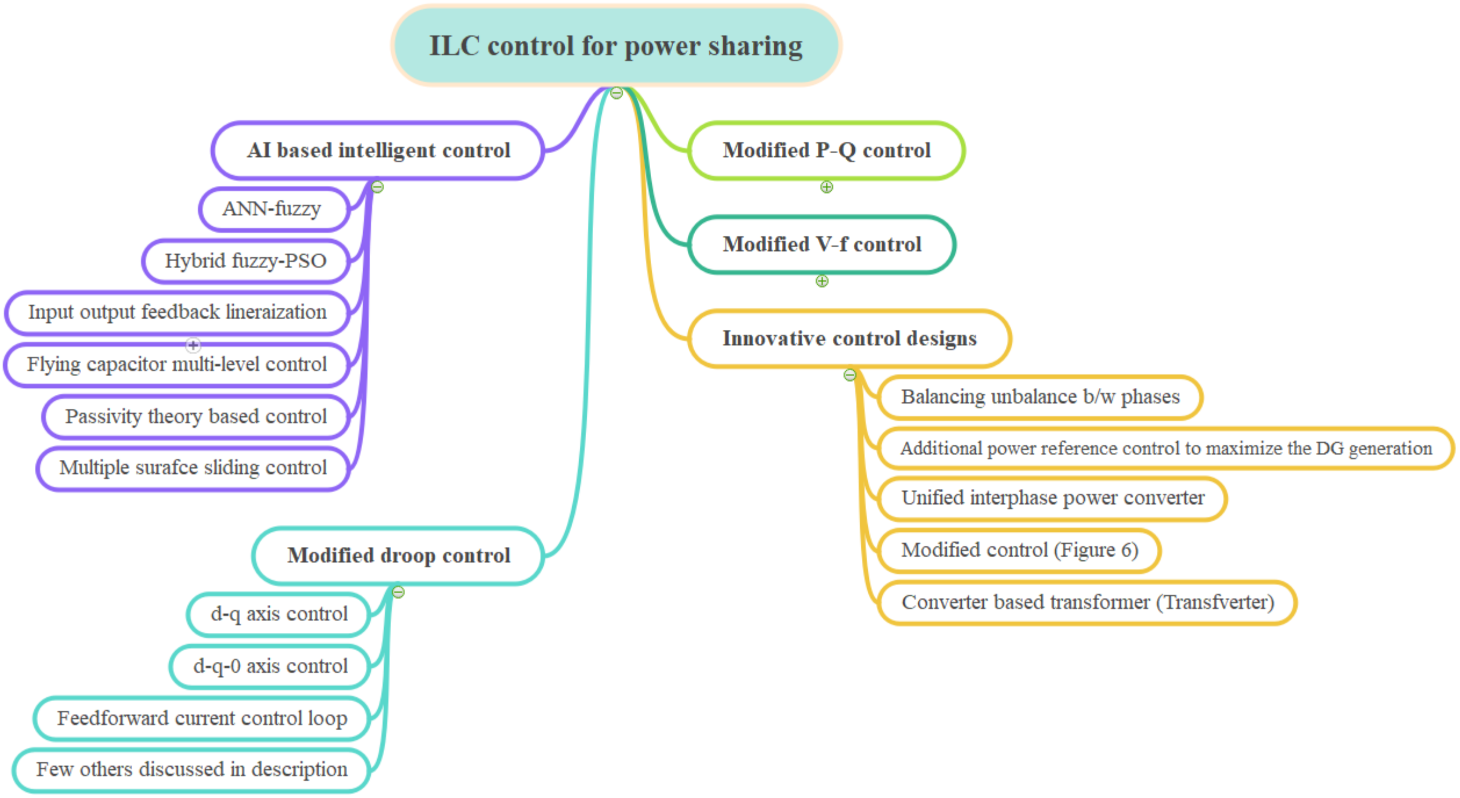

Figure 8. ILC control techniques for power sharing.

Table 3. Review table for power management of hybrid microgrid based on ILC control.

Ref	Contribution	Technique Used	Limitation	System for Validation
[43]	Basic framework for sharing power between AC and DC subgrids	Modified droop control of IC	Conventional droop control	Hybrid framework for validation is just like a generation plant with AC and DC units
[58]	Multiple nano-grids control Modified droop characteristics	$P_{dc} - v_{dc}^2$ droop control strategy	Power sharing is based on active power only ignoring reactive power effect	Multiple generating units as subgrid with local loads
[64]	Prototype of HMG with basic droop control	Decentralized basic droop control	Multiple assumptions were made about power flow and very basic control	Multiple DGs with ESS designed as an entitled subgrid

[65]	Improved efficiency and reliability by operating converters at near to full rating	Centralized control of converters for parallel operation	Basic control and assumed communication links	Multiple DG types and ESS connected to utility
[66]	Practical multi-level droop-controlled inverter	Secondary control to stabilize the voltage	Focused on active power-sharing	Only PV and ESS as DGs connected with the grid
[67]	P-f and P-v droop control for active power sharing	3D droop control	Active power sharing only	Basic HMG with 2 loads and DGs on each subgrid
[68]	Improved stability of the system with optimized power sharing through parallel ILCs	Modified droop control	Active power sharing only	ESS as DGs on both sides with increased capacity
[70]	Prototype of practical HMG	Reactive power compensation-based control of HMG	Frequency undershoot for such a small system is quite high, which may reach instability for a larger system	A DG on AC and PV on DC subgrid along with ESS
[71]	Effective power sharing including reactive power	Modified mathematical-based droop coefficient	Limited validation in stable operation only, assumed subgrids with power flow only	AC and DC microgrids assumed
[72]	Modified ILC control	d-q-0 three-axis control V_{dc}^2 regulation instead of V_{dc}	Ineffective ESS discharge causing overshoot of voltage	One PV-based DG on DC and ESS on the AC side
[73]	Suppressing circulating currents and power sharing considering capacity of subgrids	*d-q-0* axis outer control P_{dc}-V_{dc}^2 and f-P_{ac} droop for inner control	Separate control algorithm for islanding and grid-connected mode	HMG with PV as DG on both subgrids and an ESS
[74]	Improved power sharing	Modified frequency droop control based on $P_{ILC} = K\left(V_{pu} - f_{ac_{pu}}\right)$	Voltage regulation and reactive power are not considered	Single DG on each subgrid with an accumulated single load
[76]	Prototype of practical HMG	Modified current control droop strategy for ILC	Limited validation on a stable HMG	Independent microgrid with ESS
[77]	Improving the power quality and increasing the power-sharing flexibility	Modified ILC control by adding an ESS in parallel through a bidirectional DC–DC half-bridge converter and a VSC	Focused more on harmonics mitigation in grid-connected mode	An HMG with a DG each on AC and DC subgrids along with a load connected to the grid.
[82]	Robust mode transition in HMG with modified control	Model bank synthesis optimization to optimize droop coefficients	Provides voltage stability but lacks uncertainty management	Grid-connected HMG with a DG to be tested on both subgrids
[85]	Minimized transients during switching due to variable loads	Decentralized integrated droop control of ILC and RESs converter control	Assumed communication link and validated for underload generation only	Lab-based HMG prototype with three DGs in AC and two in Dc subgrid with local loads
[86]	ANN-based MPPT controller with fuzzy controller power management	ANN-based converter control and fuzzy-based power management	Grid-connected operation only limited validation with just DC generation	Wind, PV, fuel cell, and ESS as DC subgrid connected with utility

[88]	Modified control of DC/AC converters	Input–output feedback linearization and sliding mode controller-based control to DC/AC converters	The interlinking bidirectional aspect is not discussed in the control	HMG with multiple DGs supplied AC grid through DC/AC converters and a major DC load
[90]	Master–slave control for improved stability	Q-Vdc and P-Q control	Very basic control and validation for modern complex systems may be questionable	Three AC subgrids connected to the same DC-generating unit
[91]	Innovative control to improve the power quality of AC voltage	Flying capacitor multilevel converter	Just a grid-connected system for multiple DC generating units	Four DC generating units are connected to grid through a single multi-leg converter
[92]	Accurate active power and DC current sharing in AC and DC subgrid, respectively	Hierarchical control of the ILC, comprising primary and secondary control layers based on distributed consensus algorithm	Power sharing in stable conditions only	Prototype HMG in lab, consisting of 3 DGs on each subgrid
[94]	Power sharing based on common bus voltage distributed secondary control	Droop control of ILCs based on common DC bus voltage	played in only stable conditions to validate with only 4 heavy loads	An HMG infrastructure composed of 4 DGs and 2 loads on each subgrid with an ESS connected to a common DC bus
[95]	Compensating variable converter impedance and loading	Error minimization using passivity theory and surface mode controller	Improves the control of converter, but power instabilities are ignored	DC-generating units synchronized with AC grid
[96]	Efficient power sharing	Set point weighting iterative learning method	Tested under stable conditions only	Wind PV and diesel generator as sources (Aichi Microgrid setup Japan)
[97,98]	Compensating unbalanced loads by balancing the power between phases	Modified bilayer distributed control of converters	Focused only to balance the power between the phases	Two AC and DC subgrids with one specifically designed with one single- and two three-phase generators
[100]	Improved voltage and frequency stability with additional secondary control	Hierarchical secondary control to stabilize the voltage and frequency	Basic primary control and no discussion about extreme contingencies	Validated on two large networks
[101]	Enhanced upper limit of maximum power transfer through ILC with voltage and frequency support	Improved normalization of voltage and frequency with a new power reference generator	Validated with ESS only, presence of actual DGs will question the scheme	ESS as DG on both sides with a lumped load
[103]	A unique model to directly connect AC and DC subgrids with a utility grid	Modified UIPC to reduce the number of power converters	Only grid-connected balanced grid is studied	No specified subgrids, just mentions the power supplied and extracted
[104]	Reduced number of power converters for variable DC voltage requirements	Modified UDFB rectifier connected in parallel with ILC	A specific solution to supply DC distributed loads that require variable voltage	A simplified HMG with wind and PV as DGs for AC and DC subgrids, respectively

4.3. Battery Management

Energy storage is a critical component of a microgrid, and its significance is amplified when considering the HMG. In HMG, each subgrid may have multiple energy storage

units (ESUs) deployed. As a result, efficient battery management can be employed to reduce grid fluctuations. A prototype of HMG with battery management via SOC equalization is proposed in [105] to mitigate the grid fluctuations. Another SOC equalization approach for ESS-dominated HMG can offer improved power sharing among the different ESS units when paired with AC frequency and DC voltage equalized droop control [106]. The practical HMG in [105] and ESS-dominated HMG in [106] realize multiple controls such as ILC and reactive power sharing control; however, it lacks application and can only be regarded as an energy storage-based HMG rather than DGs. Hence, a better power management scheme for hybrid microgrids based on MILP optimization to maximize the renewable resources' power along with supervisory control of battery charging and discharging to regulate voltage is presented [62]. For power sharing in an isolated HMG, another fuzzy-based battery SOC controller is developed [107]. A state machine algorithm uses predefined generation modes to identify the mode of operation. In the presence of DGs, there are multiple transients due to the uncertainty of renewables and variations in load. The use of an ANFIS-based battery controller can help to reduce voltage transients in an HMG [108], which ultimately enhances the system's power stability. The overuse of battery storage to manage power may reduce the battery's life and affect the system's overall performance. A decentralized approach based on fuzzy logic is presented to optimize battery utilization [109]. The ILC control is adjusted with fuzzy logic to track the power share and usage of BES in both subgrids. Although these fuzzy and ANFIS-based BES controllers enhance battery use and help stabilize power fluctuations, their applicability is limited because the former only focuses on grid-connected systems, while the latter only focuses on islanded systems, which classifies them as problem-specific solutions.

Jiang et al. [110] propose a two-layer consensus-based method for HMG economic dispatch. Multiple traditional and renewable-based generators are scheduled for the next day to achieve minimum cost, with an energy storage system absorbing or supplying surplus/inadequate power to the system for an islanded system. An isolated HMG is vulnerable to power deficiencies. A new intelligent multi-agent-based scheme is proposed in [111] based on multi-objective PSO to compensate for the power deficiencies by curtailing some loads with predefined priorities. AC, DC, and system operator agents with separate optimization interact with each other to sustain contingencies by sharing power and utilizing programmable loads. On the other hand, to maximize the benefits of microgrid operation, an advanced metering infrastructure (AMI)-based energy management scheme is proposed in [112] for islanded hybrid AC–DC microgrids. However, load shifting and multistep load shedding strategies were not covered in this article in addition to limited application due to unavailability of an advanced metering system. The authors in [113] aimed to find an optimization strategy for the energy management of islanded AC–DC microgrids; however, they did so with a fairly simple objective function and simplified constraints, ignoring realistic constraints about line loading, ESS charging/discharging, and reactive power compensation through bidirectional ILC.

The reliable control of HMG should have ESS ready at all times to support the common DC link for any fluctuations in the system to stabilize the power share. Ref. [114] use input–output feedback linearization to realize this concept. The Lyapunov theory is used to modify the charging and discharging control of ESS. The remaining converters are updated using the same technique to compensate for renewable uncertainty. However, the battery's SOC was presumed to be completely charged, and there was no set duration for charging the battery backup after each discharge. Another Lyapunov method-based approach for compensating for the noise in a communication network for a practical HMG (opal-RT simulation environment) [115] is a modified bilayer control (that requires information from local and neighboring agents only), proposed to achieve better power sharing. This scheme is validated with multiple subgrids and is able to adaptively share active power. The current control mechanisms point to a lack of reactive power sharing and, hence, the absence of voltage support. In addition to supporting common DC bus voltage, ESS can be utilized to support the inertial response of the system by modifying its

discharging control [84]. The kinetic inertia of wind turbines and the capacity of hybrid energy storage devices are utilized to improve system stability. This method, on the other hand, ignores the effect of network impedance and has a complicated control structure. Moreover, ESS location in the hybrid microgrid impacts the dynamic response of the system [116], which is not discussed in these articles. A detailed analysis of this section is summarized in Table 4, which highlights the contribution and limitations of the schemes along with details about the system used for validation.

5. Converter Control and Protection in HMG

Local power converter control and system protection are fields that are developing quickly. The established literature in these domains has been the subject of numerous reviews. To make this a comprehensive article about hybrid microgrids, a brief discussion of the literature in these disciplines is included. A critical analysis showcases the limitations and future trends of these fields.

Table 4. Review table for battery management of hybrid microgrid based on ILC control.

Ref	Contribution	Technique Used	Limitation	System for Validation
[62]	Cost optimization by maximizing the solar and wind energy	MILP optimization to minimize the cost and maximize the renewables	Ideal converter is assumed	Hybrid framework for validation is just like a generation plant with AC and DC units
[84]	Improved inertia response	Discharging control of ESS based on inertia constant of wind turbine	Compromised power-sharing with additional complex inertia control only	HMG with wind turbine PV and ESS on both subgrids
[105]	Prototype of practical HMG with SOC equalization for multiple ESUs	Modified control of converters with additional SOC equalization	Limited test case<br>No consideration of unforeseen outages and fluctuations	PV source with multiple ESUs
[106]	Effective power sharing in ESS dominated HMG by SOC equalization of individual battery units			Multiple ESS as DGs in both subgrids
[107]	Power sharing in HMG with SOC control	State machine approach for predefined state selection<br>Fuzzy controller for battery	Basic pre-defined modes of power-sharing	Isolated HMG model with wind, diesel generator, PV, and ESS
[108]	Enhances power stability by minimizing voltage transients	ANFIS-based battery controller	Only a grid-connected and stable system is assumed	Multiple generating units with local loads
[109]	Optimal battery utilization	Fuzzy-based battery controller	Problem-specific solution to enhance battery SOC usage	Two PV panels with ESS on AC and DC side
[110]	Minimizing operational cost for the next day	Consensus-based new algorithm with modified power system constraints	Real-time load and generation uncertainty is not discussed	Four DGs and loads on each subgrid with on ILC
[111]	Optimized energy management with effective load curtailment and V2G technology	Multi-agent optimization based on multi-objective PSO	Multi-step load curtailments may cause protection to operate<br>Heavily reliance on advanced communication	Modified IEEE 33-bus system

[112]	Optimized energy management	Advanced metering and multi-objective PSO	Limited application due to the absence of advanced meters	Modified IEEE 33-bus system
[114]	Power sharing compensation for the uncertainty of renewables	Lyapunov theory and input–output feedback linearization	No schedule for battery charging	HMG with multiple renewable-based DGs
[115]	Compensating for the noise in the communication network for adaptive power-sharing	Lyapunov theory with martingale convergence theorem	Missing voltage support and reactive power sharing	Multiple nano-grid clusters

5.1. Local and Global Control of DGs and Power Converters

Power sharing in a synchronized grid equipped with multiple generation sources helped the utilities to build such a large network to supply countries and continents. Controlling their power to ensure the generator and system stability has been a concern for decades. This control becomes increasingly more difficult in a hybrid microgrid, where DC and AC DGs are coupled. Droop control has been utilized to satisfy the purpose. As science progressed, so did this technology, and numerous advanced strategies were published in the literature in order to develop a reliable and effective procedure. Multiple control techniques for generators and power converters in AC, DC, and hybrid microgrids are studied and reviewed in [14,16]. Control techniques are classified as centralized, decentralized, and distributed control in [14]. All of the analysis is based on this classification. Droop control techniques in AC microgrid for primary control framework can be classified as listed below:

- Conventional *PQ* or *v-f* control,
- *P-f* and *Q-v* control,
- Voltage real power droop/frequency-reactive power boost droop control,
- Reactive power differential of voltage *Q-V* droop,
- Angle droop control,
- Virtual frame transformation control,
- Virtual impedance-based control,
- Adaptive droop control (static and transient/dynamic droop gains),
- Unbalanced power flow control for nonlinear load sharing.

DC control can also be classified as constant voltage control, constant current control, mode-adaptive droop control [117], and modified DC droop control [16]. In addition to DG power controllers, global control at the microgrid level is appealing and requires a significant amount of research. Global control strategies can be categorized as shown in Figure 9.

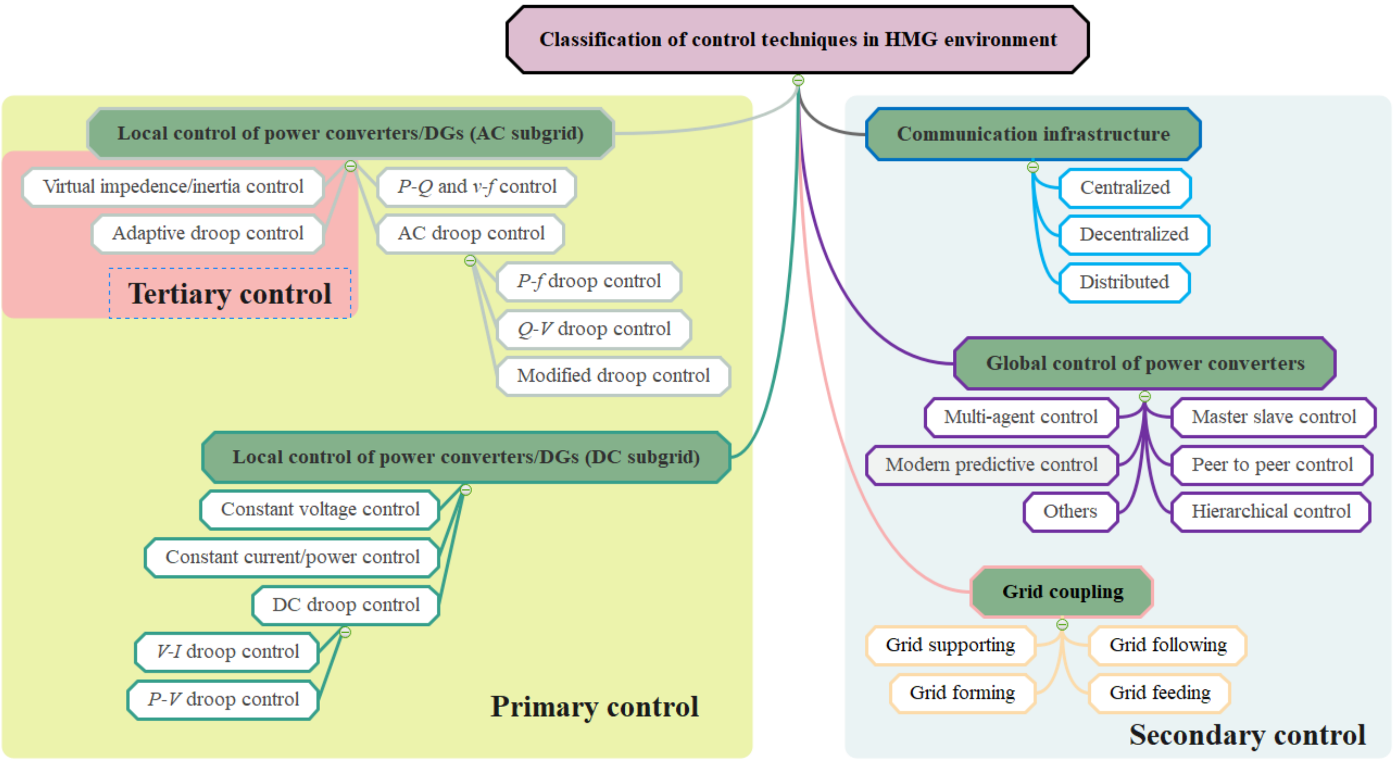

Figure 9. Global control strategies for microgrids.

The majority of the researchers in the literature have focused on converter droop control. There has been little research into PQ and *v-f* control. Hence, with the advancement in technology, this control can be modified to address the difficulties that utilities are currently facing. On the other hand, at the global level of control, the increased complexity induced by communication channels, cyber security, and dominance of low inertia inverter-based DGs prove to be problematic towards the immunity of a stable power system (microgrid).

Optimization of Controller Parameters

Microgrids have emerged as an unavoidable answer in the world of escalating energy demand and the adoption of distributed generation for a number of reasons, including increased reliability, decreased carbon footprints, and smart energy systems. The optimization of controller parameters to provide efficient load sharing and cost-effective solutions has developed into a growing area of research with the goal of improving the performance, efficiency, and cost-effectiveness of microgrids [118]. The system performance can be enhanced by identifying suitable controller parameter gains [119]. Additionally, the system will continue to run smoothly in the case of a disruption because of the optimized tuning of these parameters [120]. For instance, droop controller gains were tuned using the artificial fish swarm algorithm (AFSA) to regulate the frequency variation of an island microgrid [121]. For the automatic generation control of an interconnected system made up of renewable energy sources, a whale optimization algorithm (WOA)-based proportional integral derivative (PID) controller was presented in [122]. Similar to this, references [123] and [124] illustrate the use of WOA in the construction of proportional-integral (PI) controllers for estimating the parameters of single- and multiple-diode photovoltaic models in order to enhance the performance of solar power systems. In order to reach its undetermined properties, WOA was employed in the proton exchange membrane (PEM) fuel cell (FC) model in [125]. Additionally, [126] demonstrates the use of WOA in creating the Sugeno Fuzzy Logic controller parameters to enhance the wind power facilities' ability to ride through faults. A more enhanced whale optimization algorithm (EWOA) has

recently been created for variable-speed wind generators' maximum power point tracking (MPPT) [127].

The aforementioned methods were first created as single-objective optimization algorithms and may be termed as problem-based solutions to maximize any single objective function. Most often, though, the issues go beyond a single objective function and instead involve multiple functions that may conflict with one another, but all need to be optimized simultaneously. In another study, the controller parameters were optimized using the multi-objective based optimization algorithm known as non-dominated sorting genetic algorithm-II (NSGA-II), although no comparative analysis with other techniques was provided [128]. A novel meta-heuristic multi-objective optimization technique is developed using the hybridization of WOA with the non-dominated sorting method (NSWOA) [118]. The strength pareto evolutionary algorithm (SPEA) [129] and NSGA-II [128] (which have a comparatively slower convergence) have been used to compare and validate the performance of the proposed NSWOA. The performance analysis and statistical analysis of these methods led to the conclusion that an optimization method for controller parameters should be able to provide a stable system in a multi-mode robust microgrid with decreased overshoot and minimized oscillation frequency. In order to guarantee steady-state operations under extreme contingencies, the computing speed should also be high enough for convergence. The significance of this optimization becomes more crucial in a hybrid MG environment, where there are multiple other critical objective functions that must be optimized in addition to this for a steady-state operation.

5.2. Effect on Protection Strategies

The exponential rise of renewable-based generation in the power sector has significantly increased the inverter-based DGs in the system. The fault current contribution is limited for inverter-based DGs, especially in hybrid microgrids. The absence of neutral zero-crossing unbalanced/improper grounding will increase the chances of single-setting relay protection failure in a dual-mode microgrid [17]. Moreover, most of the protection schemes presented in the literature strongly depend on a reliable communication link, which is an ideal situation. Multiple protective schemes for HMG are analyzed in [17,18]; a short summary of the findings from these reviews with an additional critical discussion about protection of HMG is highlighted here.

In an AC subgrid, adaptive overcurrent protection requires previous knowledge of microgrid configurations, and the calculations become more complicated as the system grows larger. Adaptive differential relays are incapable of protecting the busses, also necessitating a large number of synchronizing and measuring devices [130,131]. Its performance is additionally hampered by imbalanced loading and transients. The adaptive symmetrical component-based protection technique, on the other hand, is unable to identify three-phase faults and is heavily reliant on the availability of phase-measuring devices (raising cost) and an updated grounding system [132]. Unidirectional current flows on transmission cables are protected by distance relays. However, their usefulness is debatable due to the bidirectional current flow. Distance relays are also influenced by the type of DGs and HMG configurations installed [133,134]. DC subgrid protection adds to the complexity, and reconfigurable grounding-based protection necessitates routine maintenance owing to corrosion in diode-grounded systems [135]. The DC current interruption strategy, on the other hand, shuts down the entire DC system if a fault is detected [136]. To fully protect modern hybrid systems and avoid cyber security threats, it can be concluded from the discussion above that a reliable protection scheme for HMG requires a compromised economic (higher costs of reliable communication links) and redundant protection (optimized combination of multiple traditional protections).

In addition to traditional schemes, modern protection schemes, such as pattern recognition-based schemes [137], multi-agent-based schemes, traveling wave-based schemes [138], harmonic content-based schemes [139], ANN-based schemes [140], and signal processing-based schemes [141], are presented in the most recent literature.

However, their adaptability and flexibility with changing system conditions limit their use. Moving forward, mathematical models are a suitable alternative, but handling enormous numbers of data in a timely manner can have a negative impact on the overall system. According to studies, accurate LG fault detection and high frequency are important areas of concern in reconfigurable grounding systems for DC. The smooth operation of a DC grid system requires adequate calibration of equipment and protective devices such as fuses and CBs. Existing analysis and observations of traditional methods for AC, DC, and AC/DC systems should be blended with modern advanced technologies to design and construct a robust, adaptive, and intelligent protection scheme. A practical and advanced protection strategy should have increased controllability, less reliance on the communication infrastructure [142], and operational flexibility in both grid-connected and independent modes of operation [18,143].

6. Discussion and Future Trends

The attempt to realize the concept of a hybrid microgrid has expedited significantly. As the design aspect, a fast-converging power flow analysis scheme for HMG ticking all the qualities listed in Table 1 is yet to be proposed. However, the generic method suggested in [41] solves a number of issues in the power flow analysis of an HMG. However, for an unbalanced multi-grounded hybrid microgrid with bipolar DC, this problem still requires a method to simultaneously investigate load models, voltage and frequency stability due to load variation, and renewables uncertainties. On the other hand, most power sharing/energy management efforts in the literature are focused on modifying the control architecture of HMG, especially of ILC. As a fast-growing field of research, HMG control has evolved from a single-stage primary control to multi-level hierarchical control. Figure 10 shows the primary, secondary, and tertiary controls in an HMG's hierarchical control architecture. Primary control involves current/voltage regulations and preliminary power-sharing control [144], whereas secondary control objectives may be power quality, voltage compensation, and grid support [145,146]. The top level of the hierarchical design, known as tertiary control, is concerned with cost optimization [147], dynamic load management [148], and power and energy management during unforeseen circumstances [149,150]. As you climb the control hierarchy, the control bandwidth decreases and the response time lengthens. A detailed classification of hierarchical control techniques discussed in this review is shown in Figure 10. For most applications in a power system, primary control is a low-level control and well-researched field [146]. However, a modified primary control is necessary due to the HMG's complicated structure, particularly for its ILC.

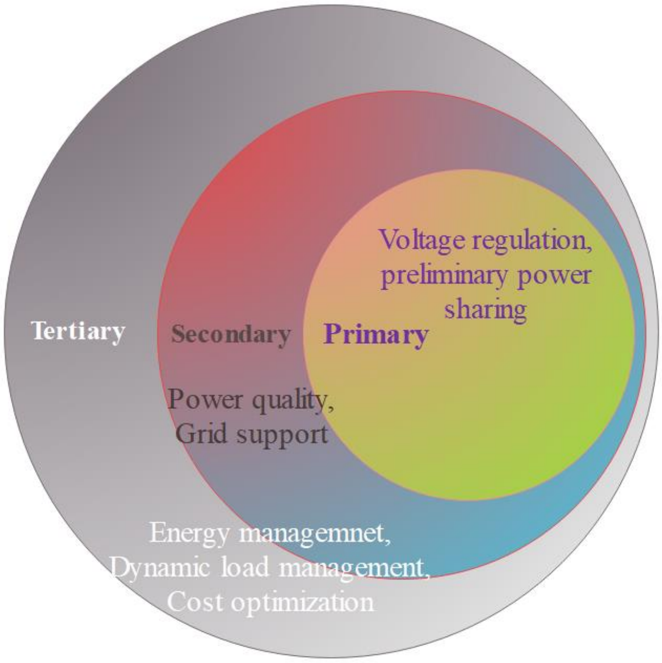

Figure 10. Classification of hierarchical control in an HMG. (Note: size of the circle shows the response time of the controller.)

The majority of the ILC control strategies presented are based on droop control such as modified droop control [43,68,71,74], multi-layer adaptive droop control [92,97,98,100], or d-q-0 axis droop control [72,73]. For stable and marginally stable systems, it provides enhanced active and reactive power management. The usefulness of these converters is questioned by the fact that decreased system inertia caused by too many power converters in the system leads to more transients and unstable circumstances, necessitating the secondary control. While one-third of the studies used artificial intelligence (AI) [86] or advanced techniques such as iterative learning with set point weighting [96] and IOFL [88] to design an adaptive control, these AI-based techniques have not been validated and/or are not feasible for a multimode test system. Although an innovative and optimized ILC control structure [100,101] can offer effective power sharing between subgrids, unplanned outages and unexpected faults might result in an unstable system. Moreover, there have been some objective-oriented, innovative control designs of ILC that serve its primary objectives, i.e., harmonics mitigation in [77], balancing power between the phases [97,98], and supplying multi-level DC to specific DC loads [104], but stabilizing the whole microgrid requires an additional control/management scheme. As a result, a supervisory control system is inevitable. A few supervisory control strategies [51,54,57,111,112] have been developed in the literature. However, most of them are dependent on predicted load and generation profiles for the following day. MILP is a widely used technique for supervisory control as more than half of the articles studied in this review adopted it for managing the next-day power, as it is the best converging technique to optimize the existing data. In addition to MILP, the modified crow search algorithm, firefly algorithm fuzzy stochastic optimization, and mathematical programming with complementary constraints are also used for the said purpose. With the advancement in AI, intelligent control techniques based on ANN-fuzzy [86], hybrid fuzzy–PSO [87], input–output feedback linearization [88], and passivity theory [95] have also been proposed. However, the implementation of these schemes in a practical environment still faces some limitations (availability of data in advance and advanced communication networks). Moreover, these have mostly only been validated on stable [87], grid-connected [86,91], or isolated systems [88]. Forecasting the day-ahead profile is a nice energy pricing strategy, but for a reliable and stable

system operation, an adaptive control scheme to address uncertainties is also required. Moreover, in a practical system, the communication of control and feedback signals is a major difficulty [115] that has been overlooked. Finally, to be deemed a robust technique, a well-designed strategy should be validated in a large enough system such as in [112]. All of the early HMG research was validated on a system that could only be described as DC-generating plants synced with the utility grid [62,65,106].

Hierarchical control (discussed above) is possible when a local converter control is used in conjunction with digital communication link-based coordinated control [151]. Dynamic decentralized or distributed control is necessary for the modern microgrid clusters to be a self-healing dynamic system. TCP/IP, P2P, PLC, UDP, and other communication protocols being developed for a microgrid have all been investigated in [152,153]. For addressing the time to send and receive information from the far end, communication time-delay standards have been established [154]. A complete blackout could occur owing to a single point of failure near the center of the system with centralized control [65] (which may be referred to as an imperfect communication infrastructure). With an increased amount of distributed generation, an information-aware distributed control [115] could energize the system (especially critical loads) during such a failure.

A variety of techniques, as discussed above, have been employed to accomplish the desired objective. Figure 11 illustrates the distribution of techniques used for ILC control and supervisory control of power between the subgrids.

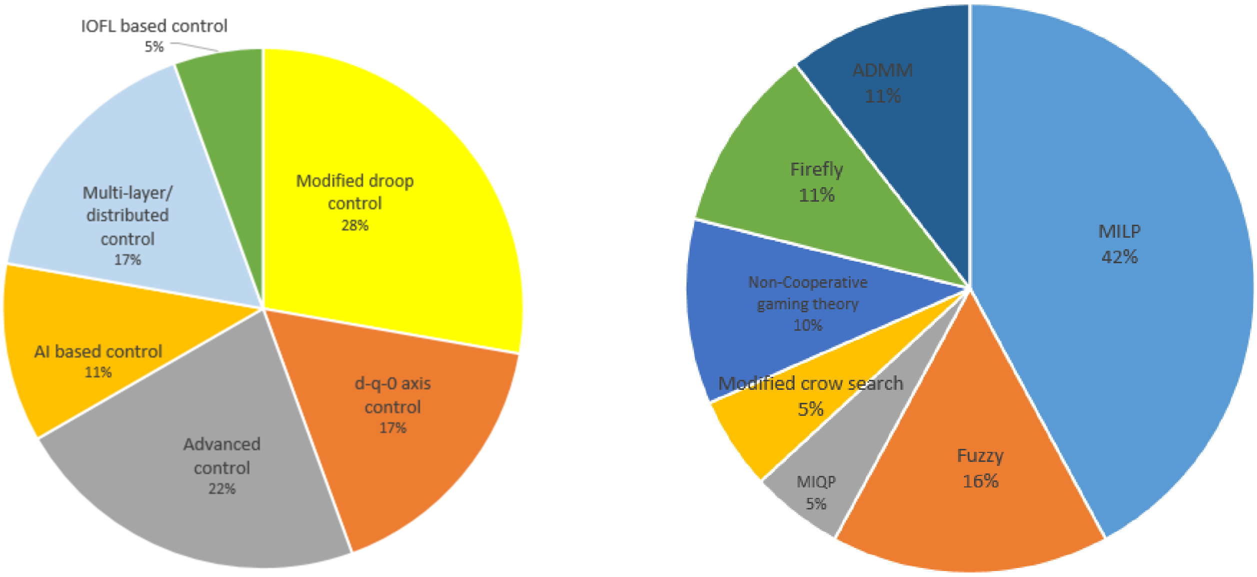

(**a**) (**b**)

Figure 11. Distribution of multiple techniques used for power management (**a**). Interlinking converter control techniques (**b**). Next-day forecasting-based supervisory control techniques.

Figure 11a explicitly shows that droop control is the most popular (30%) strategy for ILC control. The next most popular techniques are d-q-0 axis control and multi-layer control (hierarchical) methodology. The former method better manages switching transients and later accomplishes a superior reactive power sharing in addition to efficient active power sharing. Some innovative AI-based and advanced control techniques such as ANN-fuzzy [86], hybrid fuzzy–PSO [87], learning passivity theory [95], and set point weighting iterative [96] also count for 23% of the literature reviewed in this article. Similarly, MILP is the most frequently employed method for supervisory control of power, as illustrated in Figure 11b, because of its simplicity and efficient optimization of available data. Fuzzy-based and firefly algorithm-based techniques come in second and third, respectively. The advantages and limitations of all of these techniques are discussed in detail

in Sections 3 and 4. More in-depth detail about the application of these methods is presented in Tables 2–4.

Apart from the techniques used, the primary objective of a stable hybrid microgrid can be categorized into multiple sub-objectives. Figure 2 displays the distribution of several sub-objectives in this area. The most extensively studied objective is bidirectional power sharing to support adjacent subgrids in case of contingency, as expected (one-fourth of literature reviewed). The researchers' next primary objectives are to reduce costs as much as possible and manage the loading and renewable uncertainties. Only 9% of scientists made an effort to develop an HMG prototype. Surprisingly, voltage and frequency stability was not the objective of many researchers (9%). This suggests that this is a topic that future researchers should think about. With the development of technology in power electronics, the researchers have shifted their focus to adapt to advanced objectives, i.e., virtual grid support and improved power quality (harmonics). Hence, virtual grid support to generate virtual inertia and improved power quality along with voltage and frequency stability can be classified as the hot topics in this field.

6.1. Future of Hybrid Microgrids

An in-depth review of the literature on power sharing and energy management reveals that a generalized and robust scheme for optimized power sharing is yet to be developed. An operational HMG must have the following characteristics:

- Reliable and Immune: Immunity against the uncertainties of the renewables.
- Stable: Operational stability during multi-mode operation of HMG with a smooth transition of modes.
- Secure: Adaptive protection against unforeseen faults.
- Economical: Minimized cost by maximizing renewables to decarbonize the environment.

An HMG with all of these attributes simultaneously can be defined as an ideal HMG. Multiple efforts have been made to achieve these attributes in the literature. A review of these efforts in this article reveals that supervisory control is required to maximize DG power by reducing the demand and supply gap in isolated mode of operation in order to ensure the stable and reliable operation of the HMG. However, adding grid assistance in the form of virtual inertia and reactive power support while the system is connected to the grid will increase system stability. Although bidirectional power converters in HMG are well studied, it is possible to improve system reliability by modifying local unidirectional converter control to minimize transients caused by uncertainties and bidirectional power sharing. Theoretically, a global control technique to simultaneously regulate power and parameters (voltage/frequency) by centralized control of power converters appears to be considerably more efficient and effective. However, there must be major efforts made to reduce its reliance on communication infrastructure given how strongly it depends on it.

Dependence on communication is also a major hurdle in developing an effective protection scheme for a power network. Lower fault currents caused by inverter-based DGS require significant adaption, especially in islanding mode, and multi-level voltage operating in HMG makes designing a protective strategy more challenging. Last but not least, consideration must be given to the economic implications of all of these designs as well as their high-level influence on various stakeholders, including the grid operator, DNO, and users. Grid operators might need to make large capital investments to build new infrastructure. Future research may also focus on a comparative analysis of the economic benefits and environmental impact of a practical HMG, in addition to other technical research gaps outlined in this review.

Multiple research gaps are highlighted in this qualitative analysis of the short-listed literature. The following future trends in research towards HMG are listed in light of these gaps and in order to reach the attributes of the ideal HMG:

- A fast-converging power flow analysis scheme for HMG ticking all of the qualities listed in Table 1.
- Unidirectional power converters in a specified HMG environment require collaborative control (with bidirectional converters) for handling the transients in the system.
- The essential task in any electrical network (due to advancements in power electronics technology) is to reduce harmonic content and improve voltage stability (especially in power converters' dominant systems).
- Bidirectional power converters (DC–AC/DC–DC) should be modified to provide the virtual inertia and impedance to liquefy the absence (lesser number) of synchronous DGs and improved fault response, respectively, in a system dominated by the inverter-based renewable DGs. Providing voltage support (reactive power support) and frequency stability through these converters is another trending option for successfully deploying the HMG concept.
- Mathematical modeling and AI-based intelligent schemes for supervising the forecasting-based power-sharing (smart pricing) techniques to cope with uncertainties and unplanned outages is another interesting topic. The same can also be developed and utilized with an advanced control structure of power converters to mitigate the supply and demand gap for standalone operation and uncertain loading (variable demand response).
- A comprehensive under-voltage (under-frequency) load curtailment method is yet to be developed to prevent blackouts during extreme contingencies (hurricanes) and to ensure power supply for critical loads.
- The hybrid microgrid has the greatest potential for the research and development of a technique capable of protecting the system in multiple modes, particularly during mode transitions. Hence, designing an adaptive protection scheme to compensate for lower fault currents due to inverter-based DGs (especially in islanding mode) by modifying the relay setting for a multimode HMG with minimized dependence on the communication is needed.

7. Conclusions

Switching from a traditional AC or DC microgrid to an HMG substantially alters the dynamics of the system, which results in increased complexity. This article includes a critical analysis of several strategies developed to address the various challenges in a hybrid AC/DC microgrid and focused on power-sharing and energy management strategies designed specifically for a hybrid microgrid. Based on a procedural investigation of established schemes in the literature, the main conclusions can be summarized as follows: (i) there are yet only a few papers (about 15–20% of the discussed literature) that adequately validate the proposed methods on a system that complies with the term "hybrid microgrid". Hybrid suggests greater coherence between several sub-parts (multiple connections between AC and DC subgrids). (ii) More than one-fourth of the studied literature has bidirectional power sharing as its primary objective. The majority of the techniques attempted to overcome droop control's limitations. The development of power electronics calls for bidirectional converter control to evolve into a grid-supporting entity by offering reactive power support, virtual inertia, or virtual impedance during a fault. (iii) Voltage/frequency stability and power sharing can be better addressed by designing a collaborative control between global, i.e., MG control, and local, i.e., power converter control. This collaboration becomes inevitable in islanding mode to increase stability and avoid a blackout. (iv) Communication is essential to design a control system, whether it is centralized or distributed. The impact of communication technology is not a subject of many methods and is mostly assumed. (v) To increase the system's stability and dependability, it will be necessary, as in other power system areas, to combine various multi-objective control strategies for a number of power converters with centralized microgrid management plans. Along with these conclusions, this article's review tables highlight the

strengths and weaknesses of the methods deployed, and Section 6.1 outlines in detail the research gaps and potential future developments in this field.

Author Contributions: Conceptualization, S.S.; methodology, S.S. and A.E.K.; formal analysis, M.M.C.M. and D.K.; resources, S.S.; data curation, S.S.; writing—original draft preparation, S.S.; writing—review and editing, A.E.K., M.M.C.M., and D.K.; supervision, A.E.K. and M.M.C.M.; project administration, A.E.K. All authors have read and agreed to the published version of the manuscript.

Funding: This research received no external funding.

Acknowledgments: The first author would like to express his sincere gratitude to the Higher Education Commission of Pakistan for awarding a fully funded international fellowship as part of the UESTPS-UETS phase-1 HRDI faculty development program.

Conflicts of Interest: The authors declare no conflict of interest.

References

1. Mehigan, L.; Deane, J.P.; Gallachóir, B.P.Ó.; Bertsch, V. A review of the role of distributed generation (DG) in future electricity systems. *Energy* **2018**, *163*, 822–836. https://doi.org/10.1016/j.energy.2018.08.022.
2. Blaabjerg, F.; Yang, Y.; Yang, D.; Wang, X. Distributed Power-Generation Systems and Protection. *Proc. IEEE* **2017**, *105*, 1311–1331. https://doi.org/10.1109/JPROC.2017.2696878.
3. Bayod-Rújula, A.A. Future development of the electricity systems with distributed generation. *Energy* **2009**, *34*, 377–383. https://doi.org/10.1016/j.energy.2008.12.008.
4. Green, M. Community power. *Nat. Energy* **2016**, *1*, 16014. https://doi.org/10.1038/nenergy.2016.14.
5. Davis, M.W. Distributed resource electric power systems offer significant advantages over central station generation and T & D power systems. II. In Proceedings of the IEEE Power Engineering Society Summer Meeting, Chicago, IL, USA, 21–25 July 2002; Volume 61, pp. 62–69.
6. Silva, M.; Morais, H.; Vale, Z. An integrated approach for distributed energy resource short-term scheduling in smart grids considering realistic power system simulation. *Energy Convers. Manag.* **2012**, *64*, 273–288.
7. Parhizi, S.; et al., State of the art in research on microgrids: A review. *IEEE Access* **2015**, *3*, 890–925.
8. Planas, E.; Andreu, J.; Gárate, J.I.; Martínez de Alegría, I.; Ibarra, E. AC and DC technology in microgrids: A review. *Renew. Sustain. Energy Rev.* **2015**, *43*, 726–749. https://doi.org/10.1016/j.rser.2014.11.067.
9. Nejabatkhah, F.; Li, Y.W. Overview of Power Management Strategies of Hybrid AC/DC Microgrid. *IEEE Trans. Power Electron.* **2015**, *30*, 7072–7089. https://doi.org/10.1109/TPEL.2014.2384999.
10. Rocabert, J.; Luna, A.; Blaabjerg, F.; Rodríguez, P. Control of Power Converters in AC Microgrids. *IEEE Trans. Power Electron.* **2012**, *27*, 4734–4749. https://doi.org/10.1109/TPEL.2012.2199334.
11. Hossain, M.A.; Pota, H.R.; Issa, W.; Hossain, M.J. Overview of AC Microgrid Controls with Inverter-Interfaced Generations. *Energies* **2017**, *10*, 1300.
12. Whaite, S.; Grainger, B.; Kwasinski, A. Power Quality in DC Power Distribution Systems and Microgrids. *Energies* **2015**, *8*, 4378–4399.
13. Arul, P.G.; Ramachandaramurthy, V.K.; Rajkumar, R.K. Control strategies for a hybrid renewable energy system: A review. *Renew. Sustain. Energy Rev.* **2015**, *42*, 597–608. https://doi.org/10.1016/j.rser.2014.10.062.
14. Sahoo, S.K.; Sinha, A.K.; Kishore, N.K. Control Techniques in AC, DC, and Hybrid AC–DC Microgrid: A Review. *IEEE J. Emerg. Sel. Top. Power Electron.* **2018**, *6*, 738–759. https://doi.org/10.1109/JESTPE.2017.2786588.
15. Gupta, A.; Doolla, S.; Chatterjee, K. Hybrid AC–DC Microgrid: Systematic Evaluation of Control Strategies. *IEEE Trans. Smart Grid* **2018**, *9*, 3830–3843. https://doi.org/10.1109/TSG.2017.2727344.
16. Ansari, S.; Chandel, A.; Tariq, M. A Comprehensive Review on Power Converters Control and Control Strategies of AC/DC Microgrid. *IEEE Access* **2021**, *9*, 17998–18015. https://doi.org/10.1109/ACCESS.2020.3020035.
17. Mirsaeidi, S.; Dong, X.; Said, D.M. Towards hybrid AC/DC microgrids: Critical analysis and classification of protection strategies. *Renew. Sustain. Energy Rev.* **2018**, *90*, 97–103. https://doi.org/10.1016/j.rser.2018.03.046.
18. Sarangi, S.; Sahu, B.K.; Rout, P.K. Distributed generation hybrid AC/DC microgrid protection: A critical review on issues, strategies, and future directions. *Int. J. Energy Res.* **2020**, *44*, 3347–3364. https://doi.org/10.1002/er.5128.
19. Pourbehzadi, M.; Niknam, T.; Aghaei, J.; Mokryani, G.; Shafie-khah, M.; Catalão, J.P.S. Optimal operation of hybrid AC/DC microgrids under uncertainty of renewable energy resources: A comprehensive review. *Int. J. Electr. Power Energy Syst.* **2019**, *109*, 139–159. https://doi.org/10.1016/j.ijepes.2019.01.025.
20. Nejabatkhah, F.; Li, Y.W.; Tian, H. Power Quality Control of Smart Hybrid AC/DC Microgrids: An Overview. *IEEE Access* **2019**, *7*, 52295–52318. https://doi.org/10.1109/ACCESS.2019.2912376.
21. Malik, S.M.; Ai, X.; Sun, Y.; Zhengqi, C.; Shupeng, Z. Voltage and frequency control strategies of hybrid AC/DC microgrid: A review. *IET Gener. Transm. Distrib.* **2017**, *11*, 303–313. https://doi.org/10.1049/iet-gtd.2016.0791.

22. Unamuno, E.; Barrena, J.A. Hybrid ac/dc microgrids—Part I: Review and classification of topologies. *Renew. Sustain. Energy Rev.* **2015**, *52*, 1251–1259. https://doi.org/10.1016/j.rser.2015.07.194.
23. Unamuno, E.; Barrena, J.A. Hybrid AC/DC microgrids—Part II: Review and classification of control strategies. *Renew. Sustain. Energy Rev.* **2015**, *52*, 1123–1134. https://doi.org/10.1016/j.rser.2015.07.186.
24. Karabiber, A.; Keles, C.; Kaygusuz, A.; Alagoz, B.B. An approach for the integration of renewable distributed generation in hybrid DC/AC microgrids. *Renew. Energy* **2013**, *52*, 251–259. https://doi.org/10.1016/j.renene.2012.10.041.
25. Justo, J.J.; Mwasilu, F.; Lee, J.; Jung, J.-W. AC-microgrids versus DC-microgrids with distributed energy resources: A review. *Renew. Sustain. Energy Rev.* **2013**, *24*, 387–405. https://doi.org/10.1016/j.rser.2013.03.067.
26. Chandak, S.; Rout, P.K. The implementation framework of a microgrid: A review. *Int. J. Energy Res.* **2021**, *45*, 3523–3547. https://doi.org/10.1002/er.6064.
27. Yang, F.; Ye, L.; Muyeen, S.M.; Li, D.; Lin, S.; Fang, C. Power management for hybrid AC/DC microgrid with multi-mode subgrid based on incremental costs. *Int. J. Electr. Power Energy Syst.* **2022**, *138*, 107887. https://doi.org/10.1016/j.ijepes.2021.107887.
28. Tabatabaei, S.H.; Eliasi, H.; Najafi, H.; Jalilian, A. Voltage unbalancing reduction in a stand-alone ac-dc hybrid microgrid based on floating compensation reference. *Int. J. Ind. Electron. Control Optim.* **2020**, *3*, 235–247.
29. Fu, Y.; Zhang, Z.; Li, Z.; Mi, Y. Energy Management for Hybrid AC/DC Distribution System with Microgrid Clusters Using Non-Cooperative Game Theory and Robust Optimization. *IEEE Trans. Smart Grid* **2020**, *11*, 1510–1525. https://doi.org/10.1109/TSG.2019.2939586.
30. Jasemi, A.; Abdi, H. Probabilistic Multi-Objective Optimal Power Flow in an AC/DC Hybrid Microgrid Considering Emission Cost. *J. Oper. Autom. Power Eng.* **2022**, *10*, 13–27. https://doi.org/10.22098/joape.2022.8156.1565.
31. Chen, T.; Chen, M.; Hwang, K.; Kotas, P.; Chebli, E.A. Distribution system power flow analysis-a rigid approach. *IEEE Trans. Power Deliv.* **1991**, *6*, 1146–1152. https://doi.org/10.1109/61.85860.
32. Baradar, M.; Hesamzadeh, M.R.; Ghandhari, M. Second-Order Cone Programming for Optimal Power Flow in VSC-Type AC-DC Grids. *IEEE Trans. Power Syst.* **2013**, *28*, 4282–4291. https://doi.org/10.1109/TPWRS.2013.2271871.
33. Hamad, A.A.; Azzouz, M.A.; Saadany, E.F.E. A Sequential Power Flow Algorithm for Islanded Hybrid AC/DC Microgrids. *IEEE Trans. Power Syst.* **2016**, *31*, 3961–3970. https://doi.org/10.1109/TPWRS.2015.2504461.
34. Eajal, A.A.; Abdelwahed, M.A.; El-Saadany, E.F.; Ponnambalam, K. Power flow analysis of AC/DC hybrid microgrids. In Proceedings of the 2016 IEEE Electrical Power and Energy Conference (EPEC), Ottawa, ON, Canada, 12–14 October 2016; pp. 1–6.
35. Nassar, M.E.; Hamad, A.A.; Salama, M.M.A.; El-Saadany, E.F. A Novel Load Flow Algorithm for Islanded AC/DC Hybrid Microgrids. *IEEE Trans. Smart Grid* **2019**, *10*, 1553–1566. https://doi.org/10.1109/TSG.2017.2772263.
36. Baradar, M.; Ghandhari, M. A Multi-Option Unified Power Flow Approach for Hybrid AC/DC Grids Incorporating Multi-Terminal VSC-HVDC. *IEEE Trans. Power Syst.* **2013**, *28*, 2376–2383. https://doi.org/10.1109/TPWRS.2012.2236366.
37. Eajal, A.A.; Abdelwahed, M.A.; El-Saadany, E.F.; Ponnambalam, K. A Unified Approach to the Power Flow Analysis of AC/DC Hybrid Microgrids. *IEEE Trans. Sustain. Energy* **2016**, *7*, 1145–1158. https://doi.org/10.1109/TSTE.2016.2530740.
38. Ahmed, H.M.A.; Eltantawy, A.B.; Salama, M.M.A. A Generalized Approach to the Load Flow Analysis of AC–DC Hybrid Distribution Systems. *IEEE Trans. Power Syst.* **2018**, *33*, 2117–2127. https://doi.org/10.1109/TPWRS.2017.2720666.
39. Allam, M.A.; Hamad, A.A.; Kazerani, M.; El-Saadany, E.F. A steady-state analysis tool for unbalanced islanded hybrid AC/DC microgrids. *Electr. Power Syst. Res.* **2017**, *152*, 71–83. https://doi.org/10.1016/j.epsr.2017.06.027.
40. Aprilia, E.; Meng, K.; Hosani, M.A.; Zeineldin, H.H.; Dong, Z.Y. Unified Power Flow Algorithm for Standalone AC/DC Hybrid Microgrids. *IEEE Trans. Smart Grid* **2019**, *10*, 639–649. https://doi.org/10.1109/TSG.2017.2749435.
41. Pompodakis, E.E.; Kryonidis, G.C.; Demoulias, C.; Alexiadis, M.C. A Generic Power Flow Algorithm for Unbalanced Islanded Hybrid AC/DC Microgrids. *IEEE Trans. Power Syst.* **2021**, *36*, 1107–1120. https://doi.org/10.1109/TPWRS.2020.3012815.
42. Badal, F.R.; Das, P.; Sarker, S.K.; Das, S.K. A survey on control issues in renewable energy integration and microgrid. *Prot. Control Mod. Power Syst.* **2019**, *4*, 8. https://doi.org/10.1186/s41601-019-0122-8.
43. Eghtedarpour, N.; Farjah, E. Power Control and Management in a Hybrid AC/DC Microgrid. *IEEE Trans. Smart Grid* **2014**, *5*, 1494–1505. https://doi.org/10.1109/TSG.2013.2294275.
44. Shan, Y.; Hu, J.; Chan, K.W.; Fu, Q.; Guerrero, J.M. Model Predictive Control of Bidirectional DC–DC Converters and AC/DC Interlinking Converters—A New Control Method for PV-Wind-Battery Microgrids. *IEEE Trans. Sustain. Energy* **2019**, *10*, 1823–1833. https://doi.org/10.1109/TSTE.2018.2873390.
45. Teimourzadeh Baboli, P.; Shahparasti, M.; Parsa Moghaddam, M.; Haghifam, M.R.; Mohamadian, M. Energy management and operation modelling of hybrid AC–DC microgrid. *IET Gener. Transm. Distrib.* **2014**, *8*, 1700–1711. https://doi.org/10.1049/iet-gtd.2013.0793.
46. Morstyn, T.; Hredzak, B.; Aguilera, R.P.; Agelidis, V.G. Model Predictive Control for Distributed Microgrid Battery Energy Storage Systems. *IEEE Trans. Control Syst. Technol.* **2018**, *26*, 1107–1114. https://doi.org/10.1109/TCST.2017.2699159.
47. Papari, B.; Cox, R.; Sockeel, N.; Hoang, P.H.; Ozkan, G. Supervisory Energy Management in Hybrid AC-DC Microgrids Based on a Hybrid Distributed Algorithm. In Proceedings of the 2020 Clemson University Power Systems Conference (PSC), Clemson, SC, USA, 10–13 March 2020; pp. 1–6.
48. Chen, Y.; Hao, L.; Yin, G. Distributed Energy Management of the Hybrid AC/DC Microgrid with High Penetration of Distributed Energy Resources Based on ADMM. *Complexity* **2021**, *2021*, 1863855. https://doi.org/10.1155/2021/1863855.

49. Sepehrzad, R.; Moridi, A.R.; Hassanzadeh, M.E.; Seifi, A.R. Intelligent Energy Management and Multi-Objective Power Distribution Control in Hybrid Micro-grids based on the Advanced Fuzzy-PSO Method. *ISA Trans.* **2021**, *112*, 199–213. https://doi.org/10.1016/j.isatra.2020.12.027.
50. Pan, X.; Yang, F.; Ma, P.; Xing, Y.; Zhang, J.; Cao, L. A Game-Theoretic Approach of Optimized Operation of AC/DC Hybrid Microgrid Clusters. *Energies* **2022**, *15*, 5537.
51. Liang, Z.; Chen, H.; Wang, X.; Chen, S.; Zhang, C. Risk-Based Uncertainty Set Optimization Method for Energy Management of Hybrid AC/DC Microgrids With Uncertain Renewable Generation. *IEEE Trans. Smart Grid* **2020**, *11*, 1526–1542. https://doi.org/10.1109/TSG.2019.2939817.
52. Helal, S.A.; Hanna, M.O.; Najee, R.J.; Shaaban, M.F.; Osman, A.H.; Hassan, M.S. Energy Management System for Smart Hybrid AC/DC Microgrids in Remote Communities. *Electr. Power Compon. Syst.* **2019**, *47*, 1012–1024. https://doi.org/10.1080/15325008.2019.1629512.
53. Maulik, A.; Das, D. Multi-objective optimal dispatch of AC-DC Hybrid Microgrid. In Proceedings of the 2018 IEEE PES Asia-Pacific Power and Energy Engineering Conference (APPEEC), Kota Kinabalu, Malaysia, 7–10 October 2018; pp. 82–87.
54. Papari, B.; Edrington, C.S.; Bhattacharya, I.; Radman, G. Effective Energy Management of Hybrid AC–DC Microgrids With Storage Devices. *IEEE Trans. Smart Grid* **2019**, *10*, 193–203. https://doi.org/10.1109/TSG.2017.2736789.
55. Hosseinzadeh, M.; Salmasi, F.R. Fault-Tolerant Supervisory Controller for a Hybrid AC/DC Micro-Grid. *IEEE Trans. Smart Grid* **2018**, *9*, 2809–2823. https://doi.org/10.1109/TSG.2016.2620803.
56. Wang, S.; Li, L.; Huang, B.; Jiang, L. Hierarchical and decentralized optimization method based on multi-agent system for multiple AC/DC hybrid microgrids. *J. Renew. Sustain. Energy* **2018**, *10*, 045302. https://doi.org/10.1063/1.5037201.
57. Lee, J.; Kim, Y.; Moon, S. Novel Supervisory Control Method for Islanded Droop-Based AC/DC Microgrids. *IEEE Trans. Power Syst.* **2019**, *34*, 2140–2151. https://doi.org/10.1109/TPWRS.2018.2886051.
58. Xia, Y.; Wei, W.; Yu, M.; Wang, X.; Peng, Y. Power Management for a Hybrid AC/DC Microgrid With Multiple Subgrids. *IEEE Trans. Power Electron.* **2018**, *33*, 3520–3533. https://doi.org/10.1109/TPEL.2017.2705133.
59. Li, P.; Han, P.; He, S.; Wang, X. Double-uncertainty optimal operation of hybrid AC/DC microgrids with high proportion of intermittent energy sources. *J. Mod. Power Syst. Clean Energy* **2017**, *5*, 838–849. https://doi.org/10.1007/s40565-017-0336-6.
60. Loh, P.C.; Li, D.; Chai, Y.K.; Blaabjerg, F. Autonomous operation of ac–dc microgrids with minimised interlinking energy flow. *IET Power Electron.* **2013**, *6*, 1650–1657. https://doi.org/10.1049/iet-pel.2012.0567.
61. Gabbar, H.A.; El-Hendawi, M.; El-Saady, G.; Ibrahim, E.N.A. Supervisory controller for power management of AC/DC microgrid. In Proceedings of the 2016 IEEE Smart Energy Grid Engineering (SEGE), Oshawa, ON, Canada, 21–24 August 2016; pp. 147–152.
62. Hosseinzadeh, M.; Salmasi, F.R. Robust Optimal Power Management System for a Hybrid AC/DC Micro-Grid. *IEEE Trans. Sustain. Energy* **2015**, *6*, 675–687. https://doi.org/10.1109/TSTE.2015.2405935.
63. Rahmani, R.; Fakharian, A.; Guerrero, J.M. An optimal power management system for automatic connection of DC and AC resources of hybrid-microgrid systems. In Proceedings of the 2017 IEEE Second International Conference on DC Microgrids (ICDCM), Nuremburg, Germany, 27–29 June 2017; pp. 181–187.
64. Wang, P.; Jin, C.; Zhu, D.; Tang, Y.; Loh, P.C.; Choo, F.H. Distributed Control for Autonomous Operation of a Three-Port AC/DC/DS Hybrid Microgrid. *IEEE Trans. Ind. Electron.* **2015**, *62*, 1279–1290. https://doi.org/10.1109/TIE.2014.2347913.
65. Ambia, M.N.; Al-Durra, A.; Muyeen, S.M. Centralized power control strategy for AC-DC hybrid micro-grid system using multi-converter scheme. In Proceedings of the IECON 2011—37th Annual Conference of the IEEE Industrial Electronics Society, Melbourne, VIC, Australia, 7–10 November 2011; pp. 843–848.
66. Lu, X.; Guerrero, J.M.; Sun, K.; Vasquez, J.C.; Teodorescu, R.; Huang, L. Hierarchical Control of Parallel AC-DC Converter Interfaces for Hybrid Microgrids. *IEEE Trans. Smart Grid* **2014**, *5*, 683–692. https://doi.org/10.1109/TSG.2013.2272327.
67. Malik, S.M.; Sun, Y.; Huang, W.; Ai, X.; Shuai, Z. A Generalized Droop Strategy for Interlinking Converter in a Standalone Hybrid Microgrid. *Appl. Energy* **2018**, *226*, 1056–1063. https://doi.org/10.1016/j.apenergy.2018.06.002.
68. Chang, J.W.; Moon, S.I.; Lee, G.S.; Hwang, P.I. A New Local Control Method of Interlinking Converters to Improve Global Power Sharing in an Islanded Hybrid AC/DC Microgrid. *IEEE Trans. Energy Convers.* **2020**, *35*, 1014–1025. https://doi.org/10.1109/TEC.2020.2967416.
69. Worku, M.Y.; Hassan, M.A.; Abido, M.A. Real Time Energy Management and Control of Renewable Energy based Microgrid in Grid Connected and Island Modes. *Energies* **2019**, *12*, 276.
70. Ma, T.; Cintuglu, M.H.; Mohammed, O.A. Control of a Hybrid AC/DC Microgrid Involving Energy Storage and Pulsed Loads. *IEEE Trans. Ind. Appl.* **2017**, *53*, 567–575. https://doi.org/10.1109/TIA.2016.2613981.
71. Mortezapour, V.; Lesani, H. Hybrid AC/DC microgrids: A generalized approach for autonomous droop-based primary control in islanded operations. *Int. J. Electr. Power Energy Syst.* **2017**, *93*, 109–118. https://doi.org/10.1016/j.ijepes.2017.05.022.
72. Xia, Y.; Peng, Y.; Yang, P.; Yu, M.; Wei, W. Distributed Coordination Control for Multiple Bidirectional Power Converters in a Hybrid AC/DC Microgrid. *IEEE Trans. Power Electron.* **2017**, *32*, 4949–4959. https://doi.org/10.1109/TPEL.2016.2603066.
73. Yang, P.; Xia, Y.; Yu, M.; Wei, W.; Peng, Y. A Decentralized Coordination Control Method for Parallel Bidirectional Power Converters in a Hybrid AC–DC Microgrid. *IEEE Trans. Ind. Electron.* **2018**, *65*, 6217–6228. https://doi.org/10.1109/TIE.2017.2786200.
74. Peyghami, S.; Mokhtari, H.; Blaabjerg, F. Autonomous Operation of a Hybrid AC/DC Microgrid With Multiple Interlinking Converters. *IEEE Trans. Smart Grid* **2018**, *9*, 6480–6488. https://doi.org/10.1109/TSG.2017.2713941.

75. Wang, C.; Deng, C.; Li, G. Control Strategy of Interlinking Converter in Hybrid Microgrid Based on Line Impedance Estimation. *Energies* **2022**, *15*, 1664.
76. Baharizadeh, M.; Karshenas, H.R.; Guerrero, J.M. An improved power control strategy for hybrid AC-DC microgrids. *Int. J. Electr. Power Energy Syst.* **2018**, *95*, 364–373. https://doi.org/10.1016/j.ijepes.2017.08.036.
77. Carvalho Silveira, J.P.; dos Santos Neto, P.J.; dos Santos Barros, T.A.; Ruppert Filho, E. Power management of energy storage system with modified interlinking converters topology in hybrid AC/DC microgrid. *Int. J. Electr. Power Energy Syst.* **2021**, *130*, 106880. https://doi.org/10.1016/j.ijepes.2021.106880.
78. Ji, Y.; Ming, W.; Li, Y.; Wu, H. Operating Characteristic Analysis of Hybrid AC/DC Microgrid under Energy Internet. In Proceedings of the 2017 IEEE International Conference on Energy Internet (ICEI), Beijing, China, 17–21 April 2017; pp. 355–360.
79. Li, Z.; Shahidehpour, M. Small-Signal Modeling and Stability Analysis of Hybrid AC/DC Microgrids. *IEEE Trans. Smart Grid* **2019**, *10*, 2080–2095. https://doi.org/10.1109/TSG.2017.2788042.
80. Shen, X.; Tan, D.; Shuai, Z.; Luo, A. Control Techniques for Bidirectional Interlinking Converters in Hybrid Microgrids: Leveraging the advantages of both ac and dc. *IEEE Power Electron. Mag.* **2019**, *6*, 39–47. https://doi.org/10.1109/MPEL.2019.2925298.
81. Li, X.; Li, Z.; Guo, L.; Zhu, J.; Wang, Y.; Wang, C. Enhanced Dynamic Stability Control for Low-Inertia Hybrid AC/DC Microgrid With Distributed Energy Storage Systems. *IEEE Access* **2019**, *7*, 91234–91242. https://doi.org/10.1109/ACCESS.2019.2926814.
82. Gu, Y.; Li, Y.; Yoo, H.J.; Nguyen, T.T.; Xiang, X.; Kim, H.M.; Junyent-Ferre, A.; Green, T.C. Transfverter: Imbuing Transformer-Like Properties in an Interlink Converter for Robust Control of a Hybrid AC–DC Microgrid. *IEEE Trans. Power Electron.* **2019**, *34*, 11332–11341. https://doi.org/10.1109/TPEL.2019.2897460.
83. Liu, Z.; Miao, S.; Wang, W.; Sun, D. Comprehensive control scheme for an interlinking converter in a hybrid AC/DC microgrid. *CSEE J. Power Energy Syst.* **2021**, *7*, 719–729. https://doi.org/10.17775/CSEEJPES.2020.00970.
84. He, L.; Li, Y.; Guerrero, J.M.; Cao, Y. A Comprehensive Inertial Control Strategy for Hybrid AC/DC Microgrid With Distributed Generations. *IEEE Trans. Smart Grid* **2020**, *11*, 1737–1747. https://doi.org/10.1109/TSG.2019.2942736.
85. Golsorkhi, M.S.; Savaghebi, M. A Decentralized Control Strategy Based on V-I Droop for Enhancing Dynamics of Autonomous Hybrid AC/DC Microgrids. *IEEE Trans. Power Electron.* **2021**, *36*, 9430–9440. https://doi.org/10.1109/TPEL.2021.3049813.
86. Chettibi, N.; Mellit, A.; Sulligoi, G.; Pavan, A.M. Adaptive Neural Network-Based Control of a Hybrid AC/DC Microgrid. *IEEE Trans. Smart Grid* **2018**, *9*, 1667–1679. https://doi.org/10.1109/TSG.2016.2597006.
87. Maulik, A.; Das, D. Optimal power dispatch considering load and renewable generation uncertainties in an AC–DC hybrid microgrid. *IET Gener. Transm. Distrib.* **2019**, *13*, 1164–1176. https://doi.org/10.1049/iet-gtd.2018.6502.
88. Toghani Holari, Y.; Taher, S.A.; Mehrasa, M. Power management using robust control strategy in hybrid microgrid for both grid-connected and islanding modes. *J. Energy Storage* **2021**, *39*, 102600. https://doi.org/10.1016/j.est.2021.102600.
89. Ali, S.U.; Aamir, M.; Jafri, A.R.; Subramaniam, U.; Haroon, F.; Waqar, A.; Yaseen, M. Model predictive control—Based distributed control algorithm for bidirectional interlinking converter in hybrid microgrids. *Int. Trans. Electr. Energy Syst.* **2021**, *31*, e12817. https://doi.org/10.1002/2050-7038.12817.
90. Deng, W.; Pei, W.; Li, L. Active Stabilization Control of Multi-Terminal AC/DC Hybrid System Based on Flexible Low-Voltage DC Power Distribution. *Energies* **2018**, *11*, 502.
91. Sadeghi, M.; Khederzadeh, M. Hybrid multi-DC–AC MG based on multilevel interlinking converter. *IET Power Electron.* **2019**, *12*, 1187–1194. https://doi.org/10.1049/iet-pel.2018.5579.
92. Yoo, H.; Nguyen, T.; Kim, H. Consensus-Based Distributed Coordination Control of Hybrid AC/DC Microgrids. *IEEE Trans. Sustain. Energy* **2020**, *11*, 629–639. https://doi.org/10.1109/TSTE.2019.2899119.
93. Nguyen, T.H.; Van, T.L.; Nawaz, A.; Natsheh, A. Feedback Linearization-Based Control Strategy for Interlinking Inverters of Hybrid AC/DC Microgrids with Seamless Operation Mode Transition. *Energies* **2021**, *14*, 5613.
94. Nabian Dehaghani, M.; Taher, S.A.; Dehghani Arani, Z. An efficient power sharing approach in islanded hybrid AC/DC microgrid based on cooperative secondary control. *Int. Trans. Electr. Energy Syst.* **2021**, *31*, e12897. https://doi.org/10.1002/2050-7038.12897.
95. Amirkhan, S.; Radmehr, M.; Rezanejad, M.; Khormali, S. A robust control technique for stable operation of a DC/AC hybrid microgrid under parameters and loads variations. *Int. J. Electr. Power Energy Syst.* **2020**, *117*, 105659. https://doi.org/10.1016/j.ijepes.2019.105659.
96. Sendraya Perumal, A.; Kamaraj, J. Coordinated Control of Aichi Microgrid for Efficient Power Management Using Novel Set Point Weighting Iterative Learning Controller. *Energies* **2020**, *13*, 751.
97. Allam, M.A.; Hamad, A.A.; Kazerani, M.; El-Saadany, E.F. A Novel Dynamic Power Routing Scheme to Maximize Loadability of Islanded Hybrid AC/DC Microgrids Under Unbalanced AC Loading. *IEEE Trans. Smart Grid* **2018**, *9*, 5798–5809. https://doi.org/10.1109/TSG.2017.2697360.
98. Zhou, J.; Xu, Y.; Sun, H.; Li, Y.; Chow, M.Y. Distributed Power Management for Networked AC–DC Microgrids With Unbalanced Microgrids. *IEEE Trans. Ind. Inform.* **2020**, *16*, 1655–1667. https://doi.org/10.1109/TII.2019.2925133.
99. He, J.; Li, Y.W.; Blaabjerg, F. Flexible Microgrid Power Quality Enhancement Using Adaptive Hybrid Voltage and Current Controller. *IEEE Trans. Ind. Electron.* **2014**, *61*, 2784–2794. https://doi.org/10.1109/TIE.2013.2276774.
100. Abuhilaleh, M.; Li, L.; Hossain, M.J. Power management and control coordination strategy for autonomous hybrid microgrids. *IET Gener. Transm. Distrib.* **2020**, *14*, 119–130. https://doi.org/10.1049/iet-gtd.2018.5708.

101. Zheng, Q.; Gao, F. An Enhanced Control Strategy of Bidirectional Interlinking Converters in a Hybrid AC/DC Microgrid. In Proceedings of the 2021 IEEE Energy Conversion Congress and Exposition (ECCE), Vancouver, BC, Canada, 10–14 October 2021; pp. 1087–1094.
102. Eisapour-Moarref, A.; Kalantar, M.; Esmaili, M. Power sharing in hybrid microgrids with multiple DC subgrids. *Int. J. Electr. Power Energy Syst.* **2021**, *128*, 106716. https://doi.org/10.1016/j.ijepes.2020.106716.
103. Zolfaghari, M.; Abedi, M.; Gharehpetian, G.B. Power Flow Control of Interconnected AC–DC Microgrids in Grid-Connected Hybrid Microgrids Using Modified UIPC. *IEEE Trans. Smart Grid* **2019**, *10*, 6298–6307. https://doi.org/10.1109/TSG.2019.2901193.
104. Khodabakhsh, J.; Moschopoulos, G. Simplified Hybrid AC–DC Microgrid with a Novel Interlinking Converter. *IEEE Trans. Ind. Appl.* **2020**, *56*, 5023–5034. https://doi.org/10.1109/TIA.2020.2996537.
105. Brandao, D.I.; Santos, R.P.D.; Silva, W.W.A.G.; Oliveira, T.R.; Donoso-Garcia, P.F. Model-Free Energy Management System for Hybrid Alternating Current/Direct Current Microgrids. *IEEE Trans. Ind. Electron.* **2021**, *68*, 3982–3991. https://doi.org/10.1109/TIE.2020.2984993.
106. Malik, S.M.; Sun, Y. A state of charge-based linearised frequency–voltage droop for interlinking converters in an isolated hybrid microgrid. *IET Renew. Power Gener.* **2021**, *15*, 354–367. https://doi.org/10.1049/rpg2.12028.
107. Hosseinzadeh, M.; Salmasi, F.R. Power management of an isolated hybrid AC/DC micro-grid with fuzzy control of battery banks. *IET Renew. Power Gener.* **2015**, *9*, 484–493. https://doi.org/10.1049/iet-rpg.2014.0271.
108. Bhat Nempu, P.; Jayalakshmi, N.S. Coordinated Power Management of the Subgrids in a Hybrid AC–DC Microgrid with Multiple Renewable Sources. *IETE J. Res.* **2020**, *68*, 2790–2800. https://doi.org/10.1080/03772063.2020.1726829.
109. Lv, Z.; Zhang, Y.; Yu, M.; Xia, Y.; Wei, W. Decentralised coordinated energy management for hybrid AC/DC microgrid by using fuzzy control strategy. *IET Renew. Power Gener.* **2020**, *14*, 2649–2656. https://doi.org/10.1049/iet-rpg.2019.1281.
110. Jiang, K.; Wu, F.; Shi, L.; Lin, K. Distributed Hierarchical Consensus-Based Economic Dispatch for Isolated AC/DC Hybrid Microgrid. *Energies* **2020**, *13*, 3209.
111. Manbachi, M.; Ordonez, M. Intelligent Agent-Based Energy Management System for Islanded AC–DC Microgrids. *IEEE Trans. Ind. Inform.* **2020**, *16*, 4603–4614. https://doi.org/10.1109/TII.2019.2945371.
112. Manbachi, M.; Ordonez, M. AMI-Based Energy Management for Islanded AC/DC Microgrids Utilizing Energy Conservation and Optimization. *IEEE Trans. Smart Grid* **2019**, *10*, 293–304. https://doi.org/10.1109/TSG.2017.2737946.
113. Lagouir, M.; Badri, A.; Sayouti, Y. An Optimal Energy Management System of Islanded Hybrid AC/DC Microgrid. In Proceedings of the 2019 5th International Conference on Optimization and Applications (ICOA), Kenitra, Morocco, 25–26 April 2019; pp. 1–6.
114. Toghani Holari, Y.; Taher, S.A.; Mehrasa, M. Distributed energy storage system-based nonlinear control strategy for hybrid microgrid power management included wind/PV units in grid-connected operation. *Int. Trans. Electr. Energy Syst.* **2020**, *30*, e12237. https://doi.org/10.1002/2050-7038.12237.
115. Lai, J.; Lu, X.; Wang, F. Bilevel Information-Aware Distributed Resilient Control for Heterogeneous Microgrid Clusters. *IEEE Trans. Ind. Appl.* **2021**, *57*, 2014–2022. https://doi.org/10.1109/TIA.2021.3057301.
116. Daviran Keshavarzi, M.; Ali, M.H. Performance analysis of hybrid AC/DC microgrid under influence of battery energy storage location. *Int. Trans. Electr. Energy Syst.* **2021**, *31*, e13048. https://doi.org/10.1002/2050-7038.13048.
117. Gu, Y.; Xiang, X.; Li, W.; He, X. Mode-Adaptive Decentralized Control for Renewable DC Microgrid With Enhanced Reliability and Flexibility. *IEEE Trans. Power Electron.* **2014**, *29*, 5072–5080. https://doi.org/10.1109/TPEL.2013.2294204.
118. Islam, Q.N.U.; Ahmed, A.; Abdullah, S.M. Optimized controller design for islanded microgrid using non-dominated sorting whale optimization algorithm (NSWOA). *Ain Shams Eng. J.* **2021**, *12*, 3677–3689. https://doi.org/10.1016/j.asej.2021.01.035.
119. Iyer, S.V.; Belur, M.N.; Chandorkar, M.C. A Generalized Computational Method to Determine Stability of a Multi-inverter Microgrid. *IEEE Trans. Power Electron.* **2010**, *25*, 2420–2432. https://doi.org/10.1109/TPEL.2010.2048720.
120. Pogaku, N.; Prodanovic, M.; Green, T.C. Modeling, Analysis and Testing of Autonomous Operation of an Inverter-Based Microgrid. *IEEE Trans. Power Electron.* **2007**, *22*, 613–625. https://doi.org/10.1109/TPEL.2006.890003.
121. Ibrahim, A.; Jibril, Y.; Haruna, Y. Determination of Optimal Droop Controller Parameters for an Islanded Microgrid System Using Artificial Fish Swarm Algorithm (AFSA). *Int. J. Sci. Eng. Res.* **2017**, *8*, 959–965.
122. Hasanien, H.M. Whale optimisation algorithm for automatic generation control of interconnected modern power systems including renewable energy sources. *IET Gener. Transm. Distrib.* **2018**, *12*, 607–614. https://doi.org/10.1049/iet-gtd.2017.1005.
123. Hasanien, H.M. Performance improvement of photovoltaic power systems using an optimal control strategy based on whale optimization algorithm. *Electr. Power Syst. Res.* **2018**, *157*, 168–176. https://doi.org/10.1016/j.epsr.2017.12.019.
124. Elazab, O.S.; Hasanien, H.M.; Elgendy, M.A.; Abdeen, A.M. Parameters estimation of single- and multiple-diode photovoltaic model using whale optimisation algorithm. *IET Renew. Power Gener.* **2018**, *12*, 1755–1761. https://doi.org/10.1049/iet-rpg.2018.5317.
125. El-Fergany, A.A.; Hasanien, H.M.; Agwa, A.M. Semi-empirical PEM fuel cells model using whale optimization algorithm. *Energy Convers. Manag.* **2019**, *201*, 112197. https://doi.org/10.1016/j.enconman.2019.112197.
126. Qais, M.H.; Hasanien, H.M.; Alghuwainem, S. Whale optimization algorithm-based Sugeno fuzzy logic controller for fault ride-through improvement of grid-connected variable speed wind generators. *Eng. Appl. Artif. Intell.* **2020**, *87*, 103328. https://doi.org/10.1016/j.engappai.2019.103328.
127. Qais, M.H.; Hasanien, H.M.; Alghuwainem, S. Enhanced whale optimization algorithm for maximum power point tracking of variable-speed wind generators. *Appl. Soft Comput.* **2020**, *86*, 105937. https://doi.org/10.1016/j.asoc.2019.105937.

128. Wang, R.; Wu, S.; Wang, C.; An, S.; Sun, Z.; Li, W.; Xu, W.; Mu, S.; Fu, M. Optimized Operation and Control of Microgrid based on Multi-objective Genetic Algorithm. In Proceedings of the 2018 International Conference on Power System Technology (POWERCON), Guangzhou, China, 6–8 November 2018; pp. 1539–1544.
129. Zitzler, E.; Laumanns, M.; Thiele, L. *SPEA2: Improving the Strength Pareto Evolutionary Algorithm*; TIK-Report; ETH Zurich, Computer Engineering and Networks Laboratory; Zürich, Switzerland, 2001; Volume 103.
130. Sortomme, E.; Venkata, S.S.; Mitra, J. Microgrid Protection Using Communication-Assisted Digital Relays. *IEEE Trans. Power Deliv.* **2010**, *25*, 2789–2796. https://doi.org/10.1109/TPWRD.2009.2035810.
131. Kar, S.; Samantaray, S.R. Time-frequency transform-based differential scheme for microgrid protection. *IET Gener. Transm. Distrib.* **2014**, *8*, 310–320. https://doi.org/10.1049/iet-gtd.2013.0180.
132. Mirsaeidi, S.; Mat Said, D.; Mustafa, M.W.; Hafiz Habibuddin, M. A protection strategy for micro-grids based on positive-sequence component. *IET Renew. Power Gener.* **2015**, *9*, 600–609. https://doi.org/10.1049/iet-rpg.2014.0255.
133. Dewadasa, M.; Majumder, R.; Ghosh, A.; Ledwich, G. Control and protection of a microgrid with converter interfaced micro sources. In Proceedings of the 2009 International Conference on Power Systems, Kharagpur, India, 27–29 December 2009; pp. 1–6.
134. Brearley, B.J.; Prabu, R.R. A review on issues and approaches for microgrid protection. *Renew. Sustain. Energy Rev.* **2017**, *67*, 988–997. https://doi.org/10.1016/j.rser.2016.09.047.
135. Chien-Hsing, L.; Chien-Jung, L. Assessment of grounding schemes on rail potential and stray currents in a DC transit system. *IEEE Trans. Power Deliv.* **2006**, *21*, 1941–1947. https://doi.org/10.1109/TPWRD.2006.874561.
136. Tang, L.; Ooi, B.T. Locating and isolating DC faults in multi-terminal DC systems. *IEEE Trans. Power Deliv.* **2007**, *22*, 1877–1884. https://doi.org/10.1109/TPWRD.2007.899276.
137. Chakravorti, T.; Nayak, N.R.; Bisoi, R.; Dash, P.K.; Tripathy, L. A new robust kernel ridge regression classifier for islanding and power quality disturbances in a multi distributed generation based microgrid. *Renew. Energy Focus* **2019**, *28*, 78–99. https://doi.org/10.1016/j.ref.2018.12.002.
138. Li, X.; Dyśko, A.; Burt, G.M. Traveling Wave-Based Protection Scheme for Inverter-Dominated Microgrid Using Mathematical Morphology. *IEEE Trans. Smart Grid* **2014**, *5*, 2211–2218. https://doi.org/10.1109/TSG.2014.2320365.
139. Zhao, W.; Bi, X.; Wang, W.; Sun, X. Microgrid relay protection scheme based on harmonic footprint of short-circuit fault. *Chin. J. Electr. Eng.* **2018**, *4*, 64–70. https://doi.org/10.23919/CJEE.2018.8606791.
140. Lin, H.; Sun, K.; Tan, Z.-H.; Liu, C.; Guerrero, J.M.; Vasquez, J.C. Adaptive protection combined with machine learning for microgrids. *IET Gener. Transm. Distrib.* **2019**, *13*, 770–779. https://doi.org/10.1049/iet-gtd.2018.6230.
141. Abdelgayed, T.S.; Morsi, W.G.; Sidhu, T.S. A New Approach for Fault Classification in Microgrids Using Optimal Wavelet Functions Matching Pursuit. *IEEE Trans. Smart Grid* **2018**, *9*, 4838–4846. https://doi.org/10.1109/TSG.2017.2672881.
142. Erol-Kantarci, M.; Mouftah, H.T. Energy-Efficient Information and Communication Infrastructures in the Smart Grid: A Survey on Interactions and Open Issues. *IEEE Commun. Surv. Tutor.* **2015**, *17*, 179–197. https://doi.org/10.1109/COMST.2014.2341600.
143. Beheshtaein, S.; Savaghebi, M.; Vasquez, J.C.; Guerrero, J.M. Protection of AC and DC microgrids: Challenges, solutions and future trends. In Proceedings of the IECON 2015—41st Annual Conference of the IEEE Industrial Electronics Society, Yokohama, Japan, 9–12 November 2015; pp. 005253–005260.
144. Olivares, D.E.; Mehrizi-Sani, A.; Etemadi, A.H.; Cañizares, C.A.; Iravani, R.; Kazerani, M.; Hajimiragha, A.H.; Gomis-Bellmunt, O.; Saeedifard, M.; Palma-Behnke, R.; et al. Trends in Microgrid Control. *IEEE Trans. Smart Grid* **2014**, *5*, 1905–1919. https://doi.org/10.1109/TSG.2013.2295514.
145. Bidram, A.; Davoudi, A. Hierarchical Structure of Microgrids Control System. *IEEE Trans. Smart Grid* **2012**, *3*, 1963–1976. https://doi.org/10.1109/TSG.2012.2197425.
146. Gao, F.; Kang, R.; Cao, J.; Yang, T. Primary and secondary control in DC microgrids: A review. *J. Mod. Power Syst. Clean Energy* **2019**, *7*, 227–242. https://doi.org/10.1007/s40565-018-0466-5.
147. Li, C.; Bosio, F.D.; Chen, F.; Chaudhary, S.K.; Vasquez, J.C.; Guerrero, J.M. Economic Dispatch for Operating Cost Minimization Under Real-Time Pricing in Droop-Controlled DC Microgrid. *IEEE J. Emerg. Sel. Top. Power Electron.* **2017**, *5*, 587–595. https://doi.org/10.1109/JESTPE.2016.2634026.
148. Meng, L.; Dragicevic, T.; Vasquez, J.C.; Guerrero, J.M. Tertiary and Secondary Control Levels for Efficiency Optimization and System Damping in Droop Controlled DC–DC Converters. *IEEE Trans. Smart Grid* **2015**, *6*, 2615–2626. https://doi.org/10.1109/TSG.2015.2435055.
149. Li, C.; Chaudhary, S.K.; Savaghebi, M.; Vasquez, J.C.; Guerrero, J.M. Power Flow Analysis for Low-Voltage AC and DC Microgrids Considering Droop Control and Virtual Impedance. *IEEE Trans. Smart Grid* **2017**, *8*, 2754–2764. https://doi.org/10.1109/TSG.2016.2537402.
150. Zhang, Y.; Li, Y.W. Energy Management Strategy for Supercapacitor in Droop-Controlled DC Microgrid Using Virtual Impedance. *IEEE Trans. Power Electron.* **2017**, *32*, 2704–2716. https://doi.org/10.1109/TPEL.2016.2571308.
151. Boroojeni, K.; Amini, M.H.; Nejadpak, A.; Dragičević, T.; Iyengar, S.S.; Blaabjerg, F. A Novel Cloud-Based Platform for Implementation of Oblivious Power Routing for Clusters of Microgrids. *IEEE Access* **2017**, *5*, 607–619. https://doi.org/10.1109/ACCESS.2016.2646418.
152. Marzal, S.; Salas, R.; González-Medina, R.; Garcerá, G.; Figueres, E. Current challenges and future trends in the field of communication architectures for microgrids. *Renew. Sustain. Energy Rev.* **2018**, *82*, 3610–3622. https://doi.org/10.1016/j.rser.2017.10.101.

153. Wang, W.; Xu, Y.; Khanna, M. A survey on the communication architectures in smart grid. *Comput. Netw.* **2011**, *55*, 3604–3629. https://doi.org/10.1016/j.comnet.2011.07.010.
154. *IEEE Std 1646-2004*; IEEE Standard Communication Delivery Time Performance Requirements for Electric Power Substation Automation. IEEE: New York, NY, USA, 2005; pp. 1–24.
